# ConvNTC: convolutional neural tensor completion for detecting “A–A–B” type biological triplets

**DOI:** 10.1093/bib/bbaf372

**Published:** 2025-08-01

**Authors:** Pei Liu, Xiao Liang, Yue Li, Jiawei Luo

**Affiliations:** Department of Computer Science, College of Computer Science and Electronic Engineering, 116 Lu Shan South Road, Hunan University, Changsha 410082, Hunan, China; School of Computer Science, McGill University, Lorne M. Trottier Building, 3630 University Street, Montréal, QC H3A 0C6, Canada; Department of Computer Science, College of Computer Science and Electronic Engineering, 116 Lu Shan South Road, Hunan University, Changsha 410082, Hunan, China; School of Computer Science, McGill University, Lorne M. Trottier Building, 3630 University Street, Montréal, QC H3A 0C6, Canada; Department of Computer Science, College of Computer Science and Electronic Engineering, 116 Lu Shan South Road, Hunan University, Changsha 410082, Hunan, China

**Keywords:** triplet prediction, miRNA–miRNA interaction, drug combination, deep learning, tensor completion

## Abstract

Systematically investigating interactions among molecules of the same type across different contexts is crucial for unraveling disease mechanisms and developing potential therapeutic strategies. The “A–A–B” triplet paradigm provides a principled approach to model such context-specific interactions, and leveraging third-order tensor to capture such type ternary relationships is an efficient strategy. However, effectively modeling both multilinear and nonlinear characteristics to accurately identify such triplets using tensor-based methods remains a challenge. In this paper, we propose a novel **Conv**olutional **N**eural **T**ensor **C**ompletion (ConvNTC) framework that collaboratively learns the multilinear and nonlinear representations to model triplet-based network interactions. ConvNTC consists of a multilinear module and a nonlinear module. The former is a tensor decomposition approach that integrates multiple constraints to learn the tensor factor embeddings. The latter contains three components: an embedding generator to produce position-specific index embeddings for each tensor entry in addition to the factor embeddings, a convolutional encoder to perform nonlinear feature mapping while preserving the tensor’s rank-one property, and a Kolmogorov–Arnold Network (KAN) based predictor to effectively capture high-dimensional relationships aligned with the intrinsic structure of real-world data. We evaluate ConvNTC on two types triplet datasets of the “A–A–B” type: miRNA–miRNA–disease and drug–drug–cell. Comprehensive experiments against 11 state-of-the-art methods demonstrate the superiority of ConvNTC in terms of triplet prediction. ConvNTC reveals promising prognostic values of the miRNA–miRNA interactions on breast cancer and detects synergistic drug combinations in cancer cell lines.

## Introduction

Understanding the relationships between same-type biomolecules [e.g. microRNAs (miRNAs) or drugs] across different biological contexts (e.g. diseases or cell lines) is crucial for uncovering regulatory mechanisms and informing therapeutic strategies [1–3]. The A–A–B triplet framework, where A denotes molecular entities and B represents biological contexts, provides a principled approach to model such context-specific interactions. This paradigm enables the identification of synergistic or co-regulatory molecular pairs, offering insights into disease mechanisms and treatment responses. For instance, miRNA–miRNA–disease triplets reveal posttranscriptional co-regulation and aid biomarker discovery [4, 5], whereas drug–drug–disease or drug–cell line triplets facilitate the identification of effective drug combinations [6, 7].

Methodologically, the A–A–B problem can be formulated as a higher order link prediction task in heterogeneous information networks, extending traditional pairwise predictions to triplets that encode composite semantic dependencies [8, 9]. It is also closely related to subgraph matching and motif mining, where A–A–B triplets correspond to recurring substructures in heterogeneous biological graphs [10]. These motifs may reflect regulatory or therapeutic principles embedded in the network topology. Furthermore, dense subgraphs composed of A–A–B triplets often exhibit functional modularity, thus aligning the problem with community detection objectives aimed at identifying context-specific molecular modules [11–13]. Given the multiplicity of contexts (B), the A–A–B framework is naturally suited for multitask learning, where each context represents a distinct but related task. This allows for shared representation learning across molecular entities (A) while capturing context-specific interaction patterns [14, 15]. Thus, this study focuses on predicting the A–A–B triplet relationships to explore condition-specific molecular interactions.

Moreover, reformulating A–A–B triplet prediction (e.g. miRNA–miRNA–disease and drug–drug–cell/disease) as a third-order tensor completion problem has emerged as a powerful and widely adopted approach, leading to the development of various tensor-based computational models tailored to these tasks. For instance, Chen and Li [16] presented a tensor completion method, called DrugCom, for capturing disease-related drug combinations using representation learning to integrate multiple auxiliary information of drugs and diseases. Liu *et al*. [4] developed miRCom, a tensor completion framework that leverages multi-view miRNA and disease features to predict miRNA–miRNA–disease associations. Although these methods effectively identify cooperative miRNA/drug pairs, they are limited to capturing multilinear data structures, whereas real-world data often exhibit more complex nonlinear relationships rather than just multilinearity.

Subsequently, deep learning algorithms have been introduced to effectively capture nonlinear relationships. For instance, Luo *et al*. [5] developed GraphTF, a graph attention-based neural tensor factorization framework, to predict disease-associated miRNA–miRNA interactions. Although GraphTF can learn nonlinear factor embeddings through graph attention, its tensor reconstruction relies on the Kronecker product, which inherently limits its capacity to model high-order nonlinear dependencies. Sun *et al*. [7] proposed deep tensor factorization (DTF), a hybrid model integrating the classical CP_WOPT tensor decomposition with a multilayer perceptron (MLP) to predict the synergy state of drug pairs. Similarly, Han *et al*. [17] developed a novel constrained tensor factorization (CTF) integrated with a deep neural network, termed CTF-DDI, to identify multi-type drug–drug interactions. However, DTF and CTF-DDI may suffer from overfitting in sparse data settings, as they overlook the intrinsic low-rank structure of the tensor and directly feeding decomposed factors into an MLP.

Recently, the KAN framework has integrated Kolmogorov–Arnold theory with neural network architectures to enhance model capacity and interpretability [18]. Unlike traditional MLP, KAN replaces linear weight matrices with learnable B-spline functions, aiming to efficiently learn and approximate complex high-dimensional functions by decomposing them into a series of simpler functions. KAN and its derivatives have been widely applied across diverse scientific fields [19, 20]. However, to the best of our knowledge, KAN has not been effectively utilized in tensor decomposition.

In this study, we propose a hybrid deep tensor completion model, termed ConvNTC, that collaboratively captures both multilinear and nonlinear relationships to identify interactions between the same type of molecules across different biological contexts. Given the intricate and high-order dependencies inherent within a three-order tensor, two separate modules are designed for learning multilinear and nonlinear relationships. In the multilinear module, we incorporate similarity constraints, Hessian regularization, and $L_{2,1}$ regularization into a traditional tensor decomposition framework to restrict the factors learning. In contrast, the nonlinear module is designed to capture latent high-order nonlinear interactions and enhance predictive performance, which combines convolutional neural networks (CNNs) with FastKAN (a variant of KAN). This module first employs an embedding generator to obtain initial embeddings for various entities, which are subsequently processed by a convolutional encoder and a FastKAN predictor, yielding predicting scores. Extensive comparative experiments against 11 benchmark methods on two triplet prediction tasks of miRNA–miRNA–disease and drug–drug–cell demonstrate that ConvNTC exhibits notable superiority, robustness, and scalability. Additionally, case studies further illustrate the effectiveness of ConvNTC in identifying novel disease-related miRNA pairs and cell-related drug combinations.

## Materials and methods

**Figure 1:**
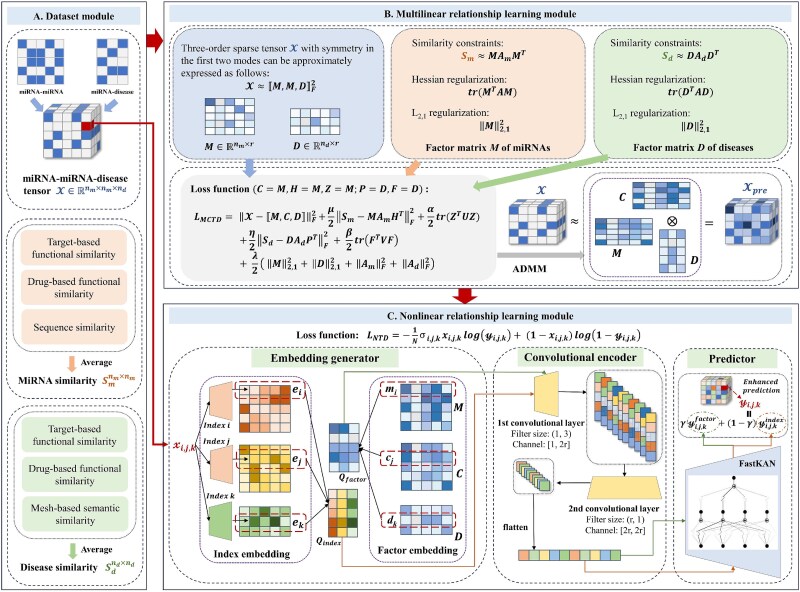
The general outline of ConvNTC by taking the predicting task of miRNA–miRNA–disease as an example. It consists of three modules: A. Dataset module. A third-order tensor $\mathcal{X}$ is constructed from the miRNA–disease and miRNA–miRNA associations to represent the miRNA–miRNA–disease triplets. The miRNA and disease similarity matrices ($S_{m}$ and $S_{d}$) are obtained by integrating multiple types of similarities using an averaging operation. B. Multilinear relationship learning module. The multilinear factor embeddings ($M$, $C$, and $D$) with $r$ components across the three dimensions of the tensor $\mathcal{X}$ are learned by introducing three constraints into a traditional tensor decomposition. C. Nonlinear relationship learning module. An embedding generator first produces the index and factor embeddings for the three dimensions corresponding to each entry $\mathcal{X}{i,j,k}$. A convolutional encoder, consisting of a two-layer convolutional network, is then designed to capture nonlinear features. Finally, a predictor based on a one-layer FastKAN is employed to further learn high-dimensional relationships and generate the enhanced prediction $\mathcal{Y}{i,j,k}$.

ConvNTC consists of three parts: the dataset module, the multilinear relationship learning module, and the nonlinear relationship learning module. ConvNTC is primarily applied to two prediction tasks i.e. miRNA–miRNA–disease and drug–drug–cell triplets. Taking “miRNA–miRNA–disease” as an example, the general outline of ConvNTC is shown in Fig. 1.

### Dataset

Details of the miRNA–miRNA–disease (MMD) and drug–drug–cell (DDC) datasets are described as follows:

- **MMD dataset.** The MMD dataset is derived from our previous work [4], containing 14 679 miRNA–miRNA–disease triplets with binary values across 351 miRNAs and 325 diseases. The assumption that all missing entries are negative samples results in a significantly larger number of negative samples compared with positive ones. To address the imbalance between positive and negative samples, an equal number of negative samples is randomly selected from the unobserved entries (except in experiments involving negative sample analysis). As a result, the dataset includes 14 679 positive samples and 14 679 negative samples.
  This dataset also includes three types of miRNA similarity (i.e. target-based functional similarity, drug-based functional similarity, and sequence similarity) and three types of disease similarity (i.e. target-based functional similarity, drug-based functional similarity, and MeSH-based similarity) [21–24]. More details on the similarity calculation methods are provided in Supplementary Material Section 3. By applying an averaging operation, the final miRNA and disease similarity matrices are obtained.
- **DDC dataset.** The drug–drug–cell synergistic triplets are collected from two publicly available oncology screening datasets: O’Neil [25] and NCI-ALMANAC [26]. For the O’Neil dataset, we directly adopt the preprocessed data from a previous study [6], which include 45 474 DDC triplets with continuous synergy scores across 38 drugs and 39 cell lines. This dataset also provides 4387 chemical descriptors for drugs and 3984 genomic features for cell lines. For the NCI-ALMANAC dataset, we use the preprocessed version provided in the referenced study [27], which consists of 148 278 DDC triplets involving 87 drugs and 55 cell lines. It includes SMILES structures for drugs and 899 gene expression profiles for cell lines. Using these features, we compute cosine similarity matrices for both drugs and cell lines.
  Since our objective is to explore synergistic interactions rather than the magnitude of synergy, the continuous synergy scores are binarized using a defined threshold. Following [6], we set the threshold to 30 to identify highly synergistic drug combinations that are of interest for clinical studies. If the synergy score of a given drug pair exceeds 30, its synergy status is set to 1 (positive); otherwise, it is set to 0 (negative). After binarization, the O’Neil dataset contains 4014 positive and 41 460 negative samples, while the NCI-ALMANAC dataset comprises 17 978 positive and 130 300 negative samples. More details are provided in Supplementary Material Section 3.

Next, we take the prediction task of miRNA–miRNA–disease triplets as an example to describe the details of other modules.

### Problem formulation

Given $n_{m}$ miRNAs, $n_{d}$ diseases, and their “A–A–B” type triplet associations $(m_{i},m_{j},d_{k})$, a third-order incomplete tensor $\mathcal{X}\in \mathbb{R}^{n_{m}\times n_{m}\times n_{d}}$ with the first two symmetric modes can be constructed, where $\mathcal{X}_{i,j,k} = 1$ represents there exists cooperative interaction between miRNA pair $(m_{i},m_{j})$ and disease $n_{d}$, and entry $\mathcal{X}_{i,j,k} = 0$ represents there is no interaction or missing value, where $i,j \in \{1,2,...,n_{m}\}$ and $k \in \{1,2,...,n_{d}\}$ are the indices of miRNA and disease, respectively. Based on this third-order tensor $\mathcal{X}$ with partially observed interactions between diseases and miRNA pairs, our purpose is to predict whether there exists potential synergistic interactions between miRNAs for a specific disease $d_{i}$ or not, by filling in the missing values of incomplete third-order tensor $\mathcal{X}$.

### Multilinear relationship learning

#### Multi-constraint tensor decomposition

Given a third-order miRNA–miRNA–disease incomplete tensor $\mathcal{X}\in \mathbb{R}^{n_{m}\times n_{m}\times n_{d}}$ with the first two symmetric modes, and two similarity matrices $S_{m}\in \mathbb{R}^{n_{m}\times n_{m}}$ and $S_{d}\in \mathbb{R}^{n_{d}\times n_{d}}$ of miRNAs and diseases, we design a multi-constraint tensor decomposition (MCTD) model, which incorporates similarity constraint, Hessian regularization, and $L_{2,1}$ regularization into the traditional INDSCAL framework [28] to enhance its overall efficacy and provide more effective model constraints. Specifically, similarity constraint can bring in additional biological information on miRNA and disease feature factors. Hessian regularization is more closely aligned with the inherent structure of data compared with conventional Laplace regularization, which can mitigate overfitting after dimensionality reduction. The $L_{2,1}$ norm enables to govern the row-wise sparsity, which can prevent overfitting and contribute to a more stable optimization process. The objective function of MCTD is formally defined as follows:


(1) \begin{align*} \mathop{L_{MCTD}} &= \Big\|\mathcal{X} -[\kern-0.15em[ M,M,D ]\kern-0.15em]\Big\|_{F}^{2} +\frac{\mu}{2}\Big\| S_{m}-M A_{m} M^{T}\Big\|_{F}^{2} \notag\\ & +\frac{\alpha}{2} {tr}\Big(M^{T} U M\Big)+\frac{\eta}{2}\Big\|S_{d}-D A_{d} D^{T}\Big\|_{F}^{2}+\frac{\beta}{2} \operatorname{tr}\Big(D^{T} V D\Big) \notag\\ & +\frac{\lambda}{2}\Big(\|M\|_{2,1}^{2}+\|D\|_{2,1}^{2}+\|A_{m}\|_{F}^{2}+\|A_{d}\|_{F}^{2}\Big),\end{align*}

where $r$ is the rank of tensor $\mathcal{X}$, $\|\cdot \|_{F}^{2}$ is the Frobenius norm, and $[\kern -0.15em[ \cdot ]\kern -0.15em]$ is the outer product operation used to reconstruct tensor; $M\in \mathbb{R}^{n_{m}\times r}, D\in \mathbb{R}^{n_{d}\times r}$ are factor matrices for miRNAs and diseases, respectively, while $A_{m},A_{d}\in \mathbb{R}^{r\times r}$ are their projection matrices; $\mu $ and $\eta $ are parameters to qualify the contribution of $S_{m}$ and $S_{d}$, respectively; $U \in \mathbb{R}^{n_{m}\times n_{m}}, V\in \mathbb{R}^{n_{d}\times n_{d}}$ are the Laplace matrices calculated from $S_{m}$ and $S_{d}$, respectively, $tr(\cdot )$ denotes the Hessian regularization term, and $\alpha ,\beta $ are parameters to control it; $\lambda $ is a parameter to control $L_{2,1}$ regularization.

#### Optimization strategy of MCTD

We employ the ADMM method as optimization strategy of MCTD. To facilitate the solution of $M$ and $D$, letting $C=M, Z=M, H=M$ and $F=D, P=D$, the augmented Lagrangian function of MCTD is constructed as follows:


(2) \begin{align*} \mathop{L_{MCTD}}&= \Big\|\mathcal{X} -[\kern-0.15em[ M,C,D ]\kern-0.15em]\Big\|_{F}^{2} + \frac{\rho_{1}}{2}\Big\|M-C+\frac{Y_{1}}{\rho_{1}}\Big\|_{F}^{2} \notag\\ & +\frac{\mu}{2}\Big\| S_{m}-M A_{m} H^{T}\Big\|_{F}^{2} + \frac{\theta_{1}}{2}\Big\|M-H+\frac{R_{1}}{\theta_{1}}\Big\|_{F}^{2} \notag\\ & +\frac{\alpha}{2} {tr}\Big(Z^{T} U Z\Big) + \frac{\rho_{2}}{2}\Big\|M-Z+\frac{Y_{2}}{\rho_{2}}\Big\|_{F}^{2} \notag\\ &+\frac{\eta}{2}\Big\|S_{d}-D A_{d} P^{T}\Big\|_{F}^{2} + \frac{\theta_{2}}{2}\Big\|D-P+\frac{R_{2}}{\theta_{2}}\Big\|_{F}^{2} \notag\\ &+\frac{\beta}{2} \operatorname{tr}\Big(F^{T} V F\Big) + \frac{\rho_{3}}{2}\Big\|D-F+\frac{Y_{3}}{\rho_{3}}\Big\|_{F}^{2} \notag\\ & +\frac{\lambda}{2}\Big(\|M\|_{2,1}^{2}+\|D\|_{2,1}^{2}+\|A_{m}\|_{F}^{2}+\|A_{d}\|_{F}^{2}\Big),\end{align*}

where $Y_{1},Y_{2},R_{1} \in \mathbb{R}^{n_{m}\times r},Y_{3},R_{2} \in \mathbb{R}^{n_{d}\times r}$ are the Lagrange multipliers; $\rho _{1},\rho _{2},\rho _{3},\theta _{1},\theta _{2}$ are the penalty parameters of them. Subsequently, each variable is updated by fixing others until convergence.

**Updating equations of $M, C, H, Z$ related to miRNAs are**


(3) \begin{align*} &M= \left(\mathcal{X}_{(1)}E+\mu S_{m} H A_{m}^{T}+ \rho_{1} C+\theta_{1} H+\rho_{2} Z - Y_{1}-R_{1}-Y_{2}\right)\notag\\ &\left(E^{T} E + \mu A_{m}H^{T}HA_{m}^{T}+\frac{\lambda}{2}W_{m}I+\rho_{1}I+\theta_{1}I+\rho_{2}I\right)^{-1} \end{align*}

(4) \begin{align*} &C= (\mathcal{X}_{(2)}G+\rho_{1} M+ Y_{1})(G^{T} G+\rho_{1}I)^{-1} \end{align*}

(5) \begin{align*} &H= (\mu S_{m} M A_{m}+ \theta_{1} M+R_{1})(\mu A_{m}M^{T}MA_{m}+\theta_{1}I)^{-1} \end{align*}

(6) \begin{align*} &Z=(\rho_{2}M+Y_{2})(\alpha U + \rho_{2}I)^{-1}, \end{align*}

where $\mathcal{X}_{(1)}$, $\mathcal{X}_{(2)}$ are the mode-1 and mode-2 matricization of tensor $\mathcal{X}$; $E=C\bigotimes D, G = M \bigotimes D$ and $\bigotimes $ is the Khatri–Rao product; $W_{m} \in \mathbb{R}^{r\times r}$ is diagonal matrix and $W_{m}(k,k)=\frac{1}{2}\|M(:,k)\|_{2}$.

**Updating equations of $D, P, F$ related to diseases are**


(7) \begin{align*} &D= (\mathcal{X}_{(3)}J+\eta S_{d} P A_{d}^{T}+\theta_{2} P+\rho_{3} F - R_{2}-Y_{3})\notag\\ &\left(J^{T} J + \eta A_{d}P^{T}PA_{d}^{T}+\frac{\lambda}{2}W_{d}I+\theta_{2}I+\rho_{3}I\right)^{-1} \end{align*}

(8) \begin{align*} &P= (\eta S_{d} D A_{d}+ \theta_{2} D+R_{2})(\eta A_{d}D^{T}DA_{d}+\theta_{2}I)^{-1} \end{align*}

(9) \begin{align*} &F=(\rho_{3}D+Y_{3})(\beta V + \rho_{3}I)^{-1}, \end{align*}

where $\mathcal{X}_{(3)}$ is the mode-3 matricization, $J=M\bigotimes C$, $W_{d} \in \mathbb{R}^{r\times r}$ is diagonal matrix, and $W_{d}(k,k)=\frac{1}{2}\|D(:,k)\|_{2}$.

**Updating equations of Lagrange multipliers $Y_{1},Y_{2},Y_{3},R_{1}$**, **and $R_{2}$ are**


(10) \begin{align*} &Y_{1}=Y_{1}+\rho_{1}(M-C), \rho_{1} = \epsilon \rho_{1} \end{align*}

(11) \begin{align*} &Y_{2}=Y_{2}+\rho_{2}(M-Z), \rho_{2} = \epsilon \rho_{2} \end{align*}

(12) \begin{align*} &Y_{3}=Y_{3}+\rho_{3}(D-F), \rho_{3} = \epsilon \rho_{3} \end{align*}

(13) \begin{align*} &R_{1}=R_{1}+\theta_{1}(M-H), \theta_{1} = \epsilon \theta_{1} \end{align*}

(14) \begin{align*} &R_{2}=R_{2}+\theta_{2}(D-P), \theta_{2} = \epsilon \theta_{2}, \end{align*}

where $\epsilon =1.15$ is the updating scale of penalty factor.

**Updating rules of projection matrices $A_{m}$ and $A_{d}$**. Taking $A_{m}$ as an example, the optimization problem of $A_{m}$ is


(15) \begin{align*} \mathop{L_{A_{m}}}=\frac{\mu}{2}\Big\| S_{m}-M A_{m} M^{T}\Big\|_{F}^{2} + \frac{\lambda}{2}\|A_{m}\|_{F}^{2}\end{align*}

To effectively update $A_{m}$, we adopt the conjugate gradient method (CG) and its updating rules ${CG}(S_{m},M,M,\mu ,\lambda )$ of $k$th iteration is


(16) \begin{align*} &a^{(k)}=\frac{\|R^{(k)}\|_{F}^{2}}{\mu\|MB^{(K)}M^{T}\|_{F}^{2} + \lambda\|B^{(k)}\|_{F}^{2}}\notag\\ &R^{(k+1)} = R^{(k)}-a^{(k)}\Big(\mu M^{T}MB^{(K)}M^{T}M +\lambda B^{(K)}\Big) \notag\\ &b^{(k)}=\frac{\|R^{(k+1)}\|_{F}^{2}}{\|R^{(k)}\|_{F}^{2}}, B^{(k+1)}=R^{(k+1)}+b^{(k)}B^{(k+)},\end{align*}

where $A_{m}^{(0)}=0, R^{(0)}=\mu M^{T}S_{m}M - \mu M^{T}MA_{m}^{(0)}M^{T}M-\lambda A_{m}^{(0)}, B^{(0)}=R^{(0)}$.

**Updating tensor $\mathcal{X}$.** Assuming that $O$ is an indicator tensor with the value of 1 and 0 to denote the corresponding position of known and unknown entries in $\mathcal{X}$, the updating formula is


(17) \begin{align*} &\mathcal{X}_{pre}= [\kern-0.15em[ M,C,D ]\kern-0.15em] \notag\\ &\hat{\mathcal{X}} = \mathcal{X} + (1-O)\mathcal{X}_{pre},\end{align*}

To this end, the learned factor matrices $M,C,D$ can be obtained until the above optimization process converges. The pseudocode of MCTD is shown in Supplementary Material Section 1.

### Nonlinear relationship learning

The core of nonlinear relationship learning module is a **N**eural Tensor Decomposition (NTD) method, which consists of three components: embedding generator, convolutional encoder, and FastKAN predictor. For a third-order incomplete tensor $\mathcal{X}\in \mathbb{R}^{n_{m}\times n_{m}\times n_{d}}$ with the first two symmetric modes, the input of NTD is an index vector $t=[i,j,k]$ and the output is an enhanced prediction score $\mathcal{Y}_{i,j,k}$.

#### Embedding generator

Given a tensor entry $\mathcal{X}_{i,j,k}$ at index $(i,j,k)$, the embedding generator takes an index vector $t=[i,j,k]$ as input, and outputs the corresponding index and factor embeddings $Q_{index},Q_{factor}$ of these indices. The index embedding of each indice is produced by a trainable operator $emb(\cdot )$ as follows:


(18) \begin{align*} &e_{i} = {emb}_{miRNA}(t[0])\in \mathbb{R}^{1\times r},t[0]=i \notag\\ &e_{j} = {emb}_{miRNA}(t[1])\in \mathbb{R}^{1\times r},t[1]=j \notag\\ &e_{k} = {emb}_{disease}(t[2])\in \mathbb{R}^{1\times r},t[2]=k \notag\\ &Q_{index} = [[e_{i}][e_{j}][e_{k}]] \in \mathbb{R}^{3\times r} \notag\\ &Q_{index} = {reshape}(Q_{index}) \in \mathbb{R}^{1\times r\times 3},\end{align*}

where $emb(\cdot ) $ is defined as $nn.Embedding$ in torch, which can map discrete inputs (i.e. miRNA and disease) to a continuous vector. Its factor embedding $Q_{factor}\in \mathbb{R}^{1\times r\times 3}$ is straightforwardly generated from the learned factors $M,C,D$.

#### Convolutional encoder

The convolutional encoder is composed of two 2D convolution layers: ${conv2d}_{1}(\cdot )$ with filter size $(1,n)$ and ${conv2d}_{2}(\cdot )$ with a filter size $(r,1)$, where $n=3$ and $r$ are the dimension and rank of association tensor, respectively. Let ${n}_{C}$ be the number of channels in the convolutional layers and $\delta _{1}=(1,n)$, $\delta _{2}=(r,1)$ are convolution filters of the two convolution layers. It should be emphasized that factor embedding $Q_{factor}$ shares the convolutional encoder with index embedding $Q_{index}$. Taking $Q_{index}$ as an example, its convolutional coding is


(19) \begin{align*} &Q_{index}^{conv1} = \sigma_{c}({{conv2d}_{1}}(Q_{index};\delta_{1})) \in \mathbb{R}^{{n}_{C}\times r\times 1}\notag\\ &Q_{index}^{conv2} = \sigma_{c}({{conv2d}_{2}}(Q_{index};\delta_{2})) \in \mathbb{R}^{{n}_{C}\times 1\times 1}\notag\\ &Q_{index}^{conv2} = {flatten}(Q_{index}^{conv2}) \in \mathbb{R}^{1\times{n}_{C}},\end{align*}

where $\sigma _{c} = ReLU(\cdot )$ is nonlinear activation function. Similarly, the convolutional coding of $Q_{factor}$ is $Q_{factor}^{conv2}\in \mathbb{R}^{1\times{n}_{C}}$.

#### FastKAN predictor

The FastKAN predictor comprises a one layer of FastKAN [29] that utilizes radial basis functions (RBFs) with Gaussian kernels to approximate third-order B-spline bases, thereby significantly accelerating model computations. More explanations are in Supplementary Material Section 2. Taking index embedding $Q_{index}^{conv2}$ as the input, the output $\mathcal{Y}_{i,j,k}^{index}$ is


(20) \begin{align*} &Q_{index}^{laynorm}=layernorm(Q_{index}^{conv2}) \in \mathbb{R}^{1\times{n}_{C}}\notag\\ &Q_{index}^{rbf}=rbf(Q_{index}^{laynorm}) \in \mathbb{R}^{{n}_{C}\times{n}_{g}}\notag\\ &Q_{index}^{rbf}=reshape(Q_{index}^{rbf}) \in \mathbb{R}^{1\times ({n}_{C}\times{n}_{g})}\notag\\ &\mathcal{Y}_{i,j,k}^{index}= w_{b} {b}(Q_{index}^{conv2}) + w_{s} Q_{index}^{rbf},\end{align*}

where $rbf(\cdot )$ denotes the Gaussian kernel-based RBF network with $n_{g}$ centers (i.e. each data point has $n_{g}$ Gaussian kernels); $layernorm(\cdot )$ is a layer normalization that ensures the input remains within a RBF domain; $b(\cdot ) = SiLU(\cdot )$ is a base activation function similar to residual connection; $w_{b},w_{s}$ are trainable factors to better control the overall magnitude of $b(\cdot )$ and $rbf(\cdot )$ functions. Similarly, the output $\mathcal{Y}_{i,j,k}^{factor}$ of factor embedding $Q_{factor}^{conv2}$ is calculated in the same way, and the final enhanced predicting score is represented as


(21) \begin{align*} \mathcal{Y}_{i,j,k}=\gamma \mathcal{Y}_{i,j,k}^{factor} + (1-\gamma) \mathcal{Y}_{i,j,k}^{index},\end{align*}

where $\gamma $ is a hyperparameter to measure the weight of index and factor embeddings. To this end, the binary cross-entropy is used to train NTD in a mini-batch way as follows:


(22) \begin{align*} L_{NTD}=-\frac{1}{N_{T}}\sum^{T}_{t} \mathcal{X}_{t}log(\mathcal{Y}_{t}) + (1-\mathcal{X}_{t})log(1-\mathcal{Y}_{t}),\end{align*}

where $N_{T}$ is the number of training entries in tensor $\mathcal{X}$, and $T \in \mathbb{R}^{N_{T}\times 3}$ is a training index matrix, and $t$ is an index vector of an entry in $\mathcal{X}$.

## Results

### Experimental setup

To systematically evaluate the performance of ConvNTC, we conducted comprehensive experiments comparing it with 11 baseline methods. Using two types triplet datasets (MMD and DDC), we performed five-fold cross-validation with five repetitions to address the following research questions:

- Can each component of ConvNTC affect the triplet prediction performance?
- Can ConvNTC outperform other state-of-the-art baseline methods across various tasks?
- Can ConvNTC discover novel and significant biological miRNA–miRNA pairs?
- Can ConvNTC prioritize potential miRNA–miRNA–disease and drug–drug–cell triplets?

A total of 11 baselines were selected for comparison with ConvNTC, including six linear models, i.e. CANDECOMP/PARAFAC (CP) [30], TFAI [31], DrugCom [16], miRCom [4], TDRC [32], and CTF [17], and five nonlinear models, i.e. DeepSynergy [6], Costco [33], DTF [7], GraphTF [5], and CTF-DDI [17]. These baselines covered both classical tensor decomposition approaches and deep learning frameworks, all designed for handling third-order incomplete tensors, and were widely applied in related biomedical association prediction tasks. The linear baselines aimed to factorize the tensor into low-rank components, with or without auxiliary information such as similarity matrices. The nonlinear baselines incorporated neural architectures to learn complex patterns and nonlinear feature interactions from the tensor or its associated inputs. By including a variety of representative methods from both model categories and applying a unified evaluation protocol, we aimed to rigorously demonstrate the effectiveness and generalizability of our proposed approach. A summary of the ConvNTC and baselines was provided in Supplementary Table S1, while their hyperparameter settings were in Supplementary Table S2.

To benchmark the predictive performance of ConvNTC, we provided seven typical metrics widely used in prediction tasks, including area under the receiver operating characteristics curve (AUROC), area under the precision–recall curve (AUPRC), F1-score, accuracy, recall, specificity, and precision. To measure the significant improvement of performance between ConvNTC and the baselines, we employed the paired t-test on these metrics at a significance level of 0.05. Notably, for experiments involving nonlinear models, 10% of the training data in each fold were further split as a validation set during the five-fold cross-validation. This validation set was used to monitor model convergence and apply early stopping when necessary. Additional details about experimental setup (including dataset division, baseline methods, evaluation metrics, and parameters setting) were provided in Supplementary Material Section 4. The parameter analysis of ConvNTC on various datasets was presented in Supplementary Material Section 5.

### Ablation study

ConvNTC comprised five key components (i.e. MCTD, index embedding, factor embedding, convolutional encoder, and FastKAN predictor) that affected its performance. To evaluate the contribution of each module within ConvNTC, we designed five variants: ConvNTC-nfact (i.e. $\gamma = 0$, no factor embedding), ConvNTC-nind (i.e. $\gamma = 1$, no index embedding), ConvNTC-nconv (i.e. no convolutional encoder), ConvNTC-mlp (i.e. FastKAN replaced with MLP), and ConvNTC-nntd (i.e. MCTD module, no NTD module). A summary of these variants is provided in Supplementary Table S3.

From the results in Table 1, we observed that ConvNTC, incorporating all five structural components, achieved the best performance on the MMD dataset and consistently ranked among the top two across the DDC datasets. These results demonstrated that removing any component led to a performance drop, highlighting their indispensable roles. Among these, ConvNTC-mlp and ConvNTC-nconv showed significant performance degradation across all datasets, indicating that FastKAN played a critical role in capturing complex high-order tensor dependencies and the convolutional encoder was essential for preserving structural patterns during nonlinear transformation. The index and factor embeddings also contributed complementary improvements, with their absence reducing precision and specificity, respectively. Furthermore, ConvNTC-nntd also showed moderate but consistent performance declines, highlighting the importance of nonlinear representation learning in capturing the high-order complex interactions among entities that cannot be fully modeled by multilinear structures alone.

**Table 1:** Performance comparison of ConvNTC and its variants across three datasets. The data in bold indicate the best performance, and those underlined indicate the second-best

Method	AUPRC	AUROC	F1	Accuracy	Recall	Specificity	Precision	Paired t-test
**MMD dataset**								
ConvNTC-mlp	0.9546	0.9151	0.9297	0.9030	0.9836	0.8223	0.9024	0.00539
ConvNTC-nconv	0.9731	0.9735	0.9290	0.9284	0.9365	0.9204	0.9218	0.00035
ConvNTC-nntd (MCTD)	0.9780	0.9706	0.9234	0.9263	0.8905	0.9621	0.9594	0.00523
ConvNTC-nind	0.9978	0.9980	0.9840	0.9839	0.9851	0.9828	0.9829	0.00379
ConvNTC-nfact	0.9982	0.9986	0.9889	0.9888	0.9925	0.9851	0.9852	0.00756
**ConvNTC**	**0.9988**	**0.9991**	**0.9926**	**0.9926**	**0.9946**	**0.9905**	**0.9906**	–
**DDC dataset (O’Neil)**								
ConvNTC-nconv	0.4208	0.8472	0.4360	0.8932	0.4665	0.9345	0.4144	0.00600
ConvNTC-mlp	0.5441	0.5000	0.1622	0.0883	**1.0000**	0.0000	0.0883	0.03030
ConvNTC-nfact	0.6945	0.9424	0.6402	0.9352	0.6528	0.9626	0.6307	0.00870
ConvNTC-nind	0.7077	0.9429	0.6518	0.9380	0.6572	0.9652	0.6490	0.01550
ConvNTC-nntd (MCTD)	**0.7625**	0.9498	**0.6997**	**0.9473**	0.6953	**0.9717**	**0.7054**	0.02290
**ConvNTC**	0.7286	**0.9503**	0.6673	0.9402	0.6796	0.9654	0.6581	–
**DDC dataset (NCI-ALMANAC)**								
ConvNTC-nconv	0.3337	0.7503	0.3761	0.8020	0.4920	0.8447	0.3054	0.00310
ConvNTC-mlp	0.3695	0.5020	0.2163	0.1212	**1.0000**	0.0000	0.1212	0.08520
ConvNTC-nfact	0.5121	0.8286	0.4923	0.8731	0.5075	0.9236	0.4807	0.00280
ConvNTC-nind	0.5475	0.8436	0.5184	0.8822	0.5231	0.9317	0.5154	0.00560
ConvNTC-nntd (MCTD)	**0.5772**	**0.8503**	**0.5437**	**0.8891**	0.5446	**0.9367**	**0.5445**	0.00790
**ConvNTC**	0.5530	0.8475	0.5220	0.8829	0.5274	0.9320	0.5179	–

Overall, these findings provided strong evidence that the collaborative learning between the multilinear structure (MCTD) and nonlinear modeling component (NTD) was critical to the success of ConvNTC. Each module contributed complementary strengths: while MCTD captured global latent factors in a low-rank manner, NTD refined the representation through context-aware nonlinear transformations, leading to superior generalization across diverse datasets. Note that we provided an extended analysis in Supplementary Material Section 6 to explain the abnormally low performance of ConvNTC-mlp on the DDC datasets. Briefly, we hypothesized that the near-random performance (AUROC around 0.5) was caused by severe class imbalance, which led the MLP-based predictor to overfit the majority class and produce near-zero predictions for all samples. Consequently, threshold-based classification yielded artificially high recall (1.0) and no discriminative power.

### Comparsion with state-of-the-art methods

#### Performance analysis

**MMD triplet dataset.** The MMD triplet dataset was constructed based on two fundamental binary associations: *miRNA–miRNA* and *miRNA–disease*. To evaluate the model’s ability to capture both higher order and pairwise biological relationships, we assessed performance under three biologically meaningful modes:

- **A–A–B (miRNA–miRNA–disease)**: direct prediction of ternary associations.
- **A–B (miRNA–disease)**: for each predicted triplet $(m_{1}, m_{2}, d)$, we derived two miRNA–disease pairs: $(m_{1}, d)$ and $(m_{2}, d)$. The prediction score from the triplet was assigned to both pairs. If a pair appeared in multiple triplets, the maximum score was retained to represent model’s confidence.
- **A–A (miRNA–miRNA)**: the miRNA pair $(m_{1}, m_{2})$ was extracted from each triplet independent of disease. For repeated pairs across triplets, the maximum score was used for evaluation.

We constructed the ground-truth labels for the A–A and A–B evaluations using the original binary association, rather than MMD triplets, to ensure that all pairwise labels reflected biologically validated knowledge and avoided label noise or information loss. Detailed derivation steps were provided in Supplementary Material Section 7. Table 2 demonstrated that our proposed ConvNTC model consistently achieved the best performance in nearly all metrics, significantly outperforming both linear and nonlinear baselines. Notably, ConvNTC exhibited superior capability in accurately capturing both higher order (A–A–B) and pairwise (A–A and A–B) relationships, as evidenced by the highest AUPRC, AUROC, and F1-score. Paired t-tests confirmed the statistical significance of the improvements. These results demonstrated the robustness and generalizability of ConvNTC for multi-relational biological inference.

**Table 2:** Performance comparison of ConvNTC and other baseline models under three evaluation modes on the MMD dataset. The data in bold indicate the best performance, and those underlined indicate the second-best

Methods		AUPRC	AUROC	F1	Accuracy	Recall	Specificity	Precision	Paired t-test
**Mode 1: A–A–B**									
Linear	TFAI	0.9283	0.8901	0.8507	0.8518	0.8439	0.8597	0.8590	0.00002
	CTF	0.9435	0.9163	0.8699	0.8737	0.8445	0.9029	0.8972	0.00014
	CP	0.9553	0.9409	0.8717	0.8734	0.8598	0.8870	0.8852	0.00027
	DrugCom	0.9682	0.9804	0.9437	0.9436	0.9457	0.9415	0.9418	0.00010
	TDRC	0.9732	0.9601	0.9238	0.9259	0.8975	0.9544	0.9518	0.00141
	**MCTD (ours)**	0.978	0.9706	0.9234	0.9263	0.8905	0.9621	0.9594	0.00523
	miRCom	0.9884	0.9826	0.9591	0.9598	0.9430	0.9767	0.9760	0.00483
Nonlinear	Costco	0.9463	0.9456	0.9077	0.9001	0.9311	0.8692	0.8914	0.00016
	GraphTF	0.9509	0.9616	0.9077	0.9059	0.9253	0.8865	0.8911	0.00022
	DeepSynergy	0.9788	0.9781	0.9274	0.9272	0.9290	0.9255	0.9259	0.00071
	CTFDDI	0.9820	0.9813	0.9305	0.9302	0.9340	0.9264	0.9271	0.00102
	DTF	0.9832	0.9836	0.9447	0.9445	0.9489	0.9401	0.9407	0.00068
	**ConvNTC (ours)**	**0.9988**	**0.9991**	**0.9926**	**0.9926**	**0.9946**	**0.9905**	**0.9906**	
**Mode 2: A–A**									
Linear	CP	0.9041	0.9400	0.8669	0.9452	0.7657	**0.9998**	**0.9993**	0.04532
	DrugCom	0.9114	0.9637	0.8468	0.9286	0.8451	0.9540	0.8492	0.00200
	TFAI	0.9148	0.9527	0.8628	0.9437	0.7599	0.9995	0.9980	0.05315
	CTF	0.9407	0.9663	0.8659	0.9372	0.8688	0.9580	0.8638	0.00240
	**MCTD (ours)**	0.9510	0.9698	0.8964	0.9541	0.8525	0.9850	0.9462	0.01066
	TDRC	0.9543	0.9674	0.9050	0.9574	0.8703	0.9839	0.9431	0.00623
	miRCom	0.9701	0.9739	0.9539	0.9792	0.9222	0.9966	0.9880	0.02625
Nonlinear	GraphTF	0.8957	0.9650	0.8462	0.9264	0.8667	0.9446	0.8276	0.00180
	Costco	0.9010	0.9429	0.8504	0.9121	0.8598	0.9284	0.8618	0.00016
	DeepSynergy	0.9373	0.9713	0.8622	0.9367	0.8488	0.9635	0.8768	0.00344
	DTF	0.9517	0.9750	0.8983	0.9530	0.8894	0.9724	0.9078	0.00252
	CTFDDI	0.9627	0.9819	0.9103	0.9583	0.9068	0.9740	0.9144	0.00323
	**ConvNTC (ours)**	**0.9952**	**0.9968**	**0.9830**	**0.9921**	**0.9770**	0.9967	0.9891	
**Mode 3: A–B**									
Linear	TFAI	0.8888	0.8863	0.8125	0.8708	0.8135	0.9009	0.8117	8.6e-05
	CP	0.8939	0.9015	0.7980	0.8668	0.7643	0.9205	0.8365	0.00022
	CTF	0.9088	0.9103	0.8353	0.8912	0.8022	0.9379	0.8720	0.00013
	DrugCom	0.9117	0.9418	0.8871	0.9251	0.8571	0.9607	0.9197	8.3e-05
	**MCTD (ours)**	0.9348	0.9366	0.8707	0.9175	0.8087	0.9746	0.9440	0.00332
	TDRC	0.9381	0.9331	0.8851	0.9253	0.8365	0.9719	0.9400	0.00069
	miRCom	0.9540	0.9480	0.9162	0.9453	0.8713	0.9840	0.9662	0.00156
Nonlinear	GraphTF	0.9068	0.9404	0.8482	0.8955	0.8493	0.9197	0.8476	0.00049
	Costco	0.9075	0.9299	0.8541	0.8877	0.8657	0.8991	0.8560	0.00024
	DeepSynergy	0.9447	0.9595	0.8769	0.9169	0.8599	0.9468	0.8947	0.00115
	CTFDDI	0.9549	0.9656	0.8887	0.9250	0.8707	0.9535	0.9078	0.00199
	DTF	0.9631	0.9733	0.9077	0.9374	0.8947	0.9598	0.9211	0.00578
	**ConvNTC (ours)**	**0.9765**	**0.9787**	**0.9507**	**0.9672**	**0.9192**	**0.9924**	**0.9845**	

**DDC triplet dataset.** We evaluated ConvNTC and various baseline models on two real-world drug–drug-cell line datasets: O’Neil and NCI-ALMANAC. Results in Supplementary Table S4 showed that ConvNTC consistently achieved the best or second-best performance across most metrics, particularly excelling in AUROC, Accuracy, and Specificity. On the O’Neil dataset, ConvNTC outperformed all baselines in AUROC (0.9503), while MCTD achieved slightly higher AUPRC. On the NCI-ALMANAC dataset, ConvNTC showed superior performance in AUROC (0.8475), Accuracy (0.8829), and Specificity (0.9320), outperforming most linear and nonlinear baselines. These results validated ConvNTC’s robust generalization ability and effectiveness in handling real-world noisy and imbalanced biomedical data.

While nonlinear baselines generally outperformed linear methods due to their ability to capture complex tensor structures, most failed to maintain consistent performance across datasets, except for DTF. This highlighted the benefit of incorporating multilinear factor embeddings to better model nonlinear dependencies. ConvNTC further surpassed DTF across nearly all metrics, validating the strength of its architectural design. Importantly, ConvNTC’s unified prediction space supported consistent downstream evaluation under A–A–B, A–A, and A–B modes, offering flexible interpretability at multiple levels of biological granularity. These findings collectively demonstrated ConvNTC’s architectural advantages and its practical value in multi-relational biomedical prediction tasks.

#### Robustness analysis

**Figure 2:**
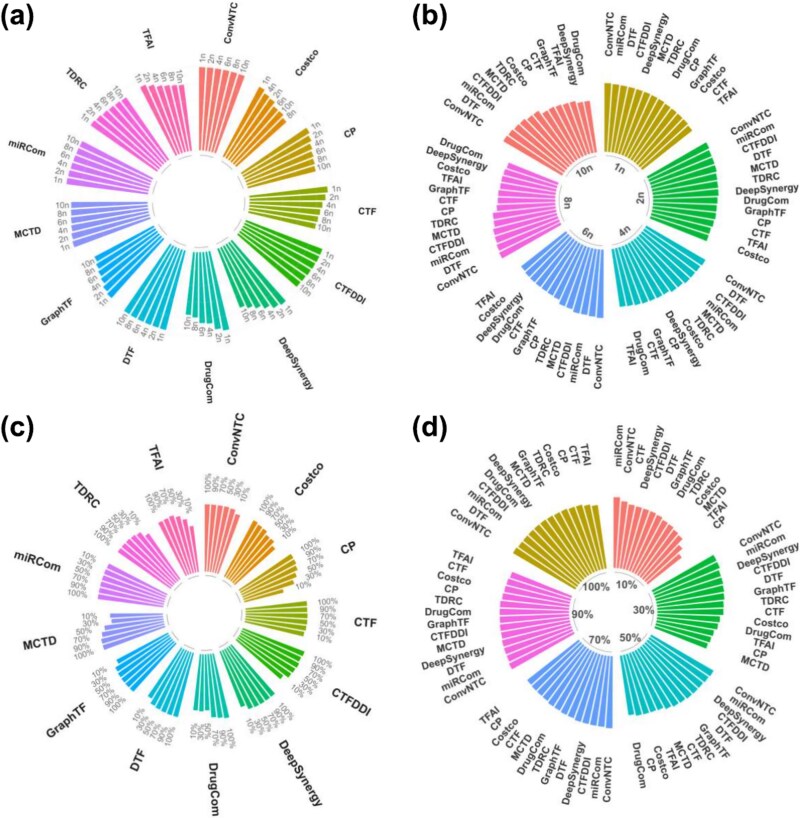
Comparison results of robustness analysis. (a) and (b) are the AUPRC values of all methods in different negative sample sizes, where n is the number of positive sample. (c) and (d) are the AUROC values of all methods in various training ratios used to train the model, where 10%, 30%, 50%, 70%, 90%, and 100% are the percentages of the original known data.

To systematically assess the robustness of ConvNTC, we explored the influence of negative sample size and training ratio on MMD dataset. We first randomly sampled the negative samples in size of $[1n,2n,4n,6n,8n,10n]$, where $n$ was the number of positive samples. Figure 2a) and b depicted the AUPRC values of ConvNTC and baselines, which indicated that all methods decreased as the negative sample size increased, and ConvNTC performed best. Additionally, the decrease in ConvNTC was significantly lower than others, indicating that ConvNTC exhibited strong robustness against imbalanced data. To assess the model performance under different training ratios, we set the training ratios to 10%, 30%, 50%, 70%, 90%, and 100% of the original known data. Figure 2c and d showed that the AUROC values of most models increased with higher training ratios, and ConvNTC consistently outperformed all other baselines, underscoring that ConvNTC exhibited resilience to variations in training ratio.

#### Generalizability analysis on unseen entities

To evaluate the generalization ability of ConvNTC, we conducted entity-level five-fold cross-validation on MMD and DDC datasets. In each setting, all associations involving a particular entity (e.g. cell line, disease, miRNA, or drug) were excluded from the training data, ensuring no information leakage. Detailed division rules were shown in Supplementary Material Section 4. Results in Supplementary Table S5 summarized model performance on unseen diseases and cells. ConvNTC achieved the best results on the MMD dataset, outperforming all baselines across all metrics. This demonstrated its strong generalization for predicting new disease-associated miRNA interaction. On the DDC datasets (O’Neil and NCI-ALMANAC), ConvNTC performed comparably with the strongest baselines. Specifically, on the O’Neil dataset, ConvNTC achieved the second-best AUROC (0.8478) and a balanced recall–specificity trade-off. DTF achieved marginally better scores in a few metrics, but ConvNTC offered more stable generalization across metrics. On the NCI-ALMANAC dataset, ConvNTC yielded the competitive overall performance in most metrics including AUROC (0.7583) and Accuracy (0.8586), confirming its robustness to unseen cellular contexts.

In addition, we explored the performance of ConvNTC and baselines under a more challenging scenario where miRNA or drug entities were entirely unseen during training. The results were showed in Supplementary Table S6. On the MMD dataset, ConvNTC lagged behind baselines such as DeepSynergy that achieved the highest AUROC and AUPRC. On the DDC-O’Neil dataset, ConvNTC ranked among the top performers in AUROC (0.7478), closely following DTF and GraphTF. Meanwhile, ConvNTC consistently surpassed all linear models in most evaluation metrics indicating its resilience. A similar trend was observed on NCI-ALMANAC dataset, where ConvNTC maintained competitive performance in most metrics against the majority of baselines.

### Investigation of the predicted miRNA–miRNA pairs

To systematically evaluate the effectiveness of the miRNA interaction network constructed in this study, we first applied the trained ConvNTC model on the MMD dataset to obtain prediction scores for all candidate miRNA–miRNA–disease triplets. For each miRNA pair, we aggregated the prediction scores across all related diseases to compute a comprehensive association score. To enhance the biological interpretability of the resulting network, self-looping miRNA pairs were removed, and the remaining pairs were ranked based on their aggregated scores. The top 0.5% of miRNA pairs were selected to construct a predicted miRNA–miRNA interaction network, comprising 203 miRNA nodes and 615 edges. The known miRNA–miRNA network, comprising 561 edges and 217 miRNA nodes, was constructed using miRNA family information from miRBase.

#### Topological properties

To assess whether the topological properties of the predicted miRNA–miRNA networks significantly deviated from random expectation, we performed a permutation-based significance test for each network-level metric (e.g. average degree, density, clustering coefficient). To establish the null distribution for each metric, we generated $ n = 1000 $ randomized networks. These were constructed by taking the union of all nodes present in both the predicted and known networks (i.e. 224 nodes), and fixing the number of edges to match that of the predicted network (i.e. 615 edges). Edges were randomly reassigned between nodes while avoiding self-loops and duplicate connections. Each randomized network was analyzed using the same topological metrics. Empirical two-sided $ P $-values were then computed by comparing each observed metric against its corresponding distribution from the random networks. To account for multiple testing across different metrics, we applied the Benjamini–Hochberg procedure to control the false discovery rate (FDR).

**Figure 3:**
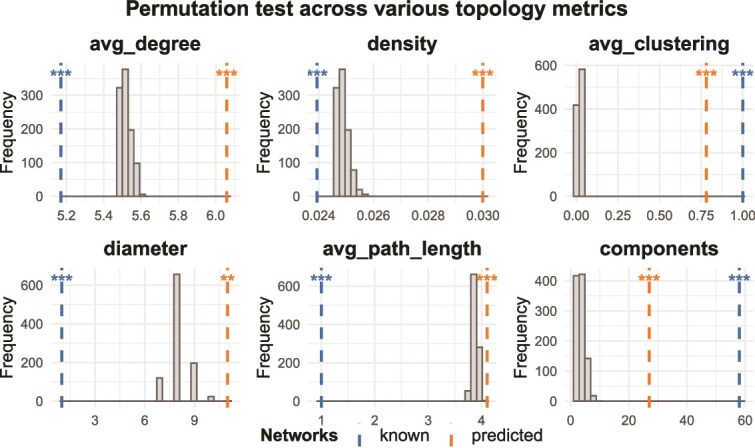
Permutation-based comparison of topological features between known/predicted miRNA–miRNA networks and 1000 random networks. Each subplot shows the distribution of a topological metric derived from random networks (gray histogram), overlaid with vertical dashed lines indicating the observed values from known (blue) and predicted (orange) networks. Statistical significance is annotated above each line using FDR-corrected thresholds (^*^ for FDR $\leq $ 0.05, ^*^^*^ for FDR $\leq $ 0.01, ^*^^*^^*^ for FDR $\leq $ 0.001).

As shown in Fig. 3, the permutation tests against 1000 random networks confirmed that both known and predicted networks significantly deviated from random topology across all metrics (FDR $\leq $ 0.05). Notably, the predicted network displayed higher average degree and density compared with the known network, suggesting that the predicted interactions formed a denser connectivity pattern. In contrast, the known network showed shorter diameter and path length, indicating greater global compactness. These differences highlighted that the predicted network not only retained nonrandom topological features similar to the known network but also introduced additional topological complexity, potentially reflecting novel interactions inferred by ConvNTC.

In addition, we compared both the predicted and the first random miRNA–miRNA network against the known one, and found that the predicted network recovered 446 known interactions and identified 169 novel associations, whereas the random network contained only 12 known interactions Their overlaps are shown in Supplementary Figure S1. Notably, all miRNAs in 169 novel associations from the predicted network were different families, as family-based miRNA–miRNA interactions were explicitly used in the model training. This comparison demonstrated that our model not only accurately recovered known miRNA interactions but also exhibits strong potential to uncover novel, functionally relevant miRNA–miRNA associations.

#### Functional similarity

To evaluate the biological plausibility of the predicted novel miRNA interactions, we performed a comprehensive functional similarity analysis of miRNA pairs from both the predicted and random networks across four biological annotation types: Disease, Gene Ontology Biological Process (GOBP), Reactome, and WikiPathway. Three metrics were used to quantify functional similarity: the number of shared annotations, Jaccard similarity, and enrichment significance score.

For the Disease annotation type, we constructed a unified miRNA–disease association network by integrating data from HMDD v4, HMDD v3.2, PhenomiR, and miRCancer databases. For each miRNA pair, we calculated the number of shared associated diseases and the corresponding Jaccard similarity to measure their co-regulation at the disease level. For the GOBP/Reactome/WikiPathway annotation types, enrichment analyses were performed for each miRNA’s target genes using high-confidence miRNA-target associations from miRTarBase. Only significantly enriched terms (adjusted $P <0.05$) were retained. Based on these enrichment results, we calculated both the number of shared enriched terms and the Jaccard similarity for each miRNA pair within each functional category. Furthermore, to assess the statistical significance of functional overlap between miRNA pairs, we applied a hypergeometric test to determine whether the observed number of shared annotations was greater than expected by chance. This test was conducted for each pair across all annotation types and provided additional evidence supporting the functional relevance of the predicted miRNA–miRNA interactions.

**Figure 4:**
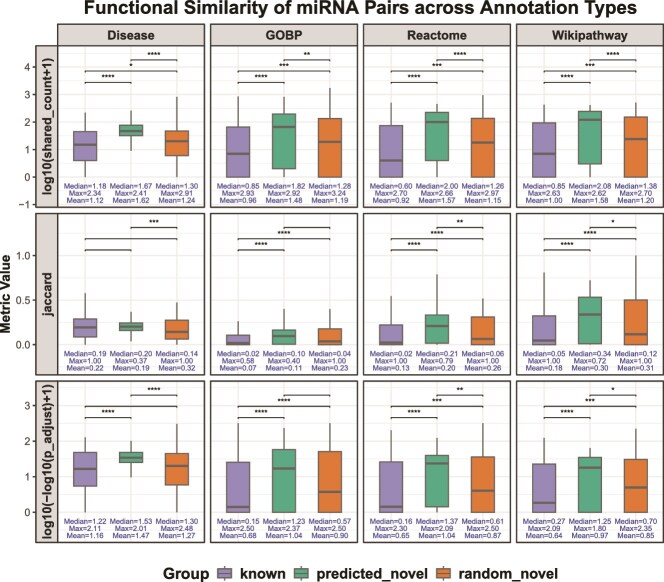
Distributions of functional similarity metrics across four annotation types for three categories of miRNA pairs: *known*, *predicted_novel* (novel edges in predicted network), and *random_novel* (novel edges in random network). Statistical significance between groups is indicated using asterisks, where more asterisks represent stronger significance. Notably, log10(shared_count + 1) reflects shared annotations, and log10(-log10(p_adjusted) + 1) represents enrichment significance.

As illustrated in Fig. 4, we observed that the functional similarity scores of the predicted novel miRNA–miRNA interactions were always significantly higher than those of random pairs, and in most cases, they were able to obtain functional similarity distributions comparable with those of known pairs. Notably, the predicted novel pairs achieved the highest median values across nearly all metrics and annotation types. This trend was particularly evident in Reactome and WikiPathway, where the predicted interactions significantly outperformed both the known and random groups. These observations indicated that the predicted miRNA interactions were not random occurrences, but rather reflected underlying biological coherence. This further highlighted the capacity of the ConvNTC model to uncover biologically meaningful but previously unreported miRNA–miRNA interactions.

#### Classification and analysis of the novel miRNA pairs

To validate the biological significance of the predicted novel miRNA–miRNA pairs, we first performed a functional classification based on the above functional similarity of four annotation types. Each miRNA pair was categorized based on disease co-association and shared functional pathways. Specifically, pairs were labeled as “ubiquitous” if they shared more diseases than the dataset median and also had at least one shared pathway in Reactome, WikiPathway, or GOBP. Those with fewer or equal disease overlaps but with shared functional pathways were labeled as “disease-specific.” Pairs without any shared functional pathway were grouped as “other.”

To further strengthen the classification, we introduced an expression-based strategy using precursor miRNA expression profiles across 33 cancer types from TCGA. For each miRNA pair, we calculated Pearson correlation coefficients across cancers and identified those showing high absolute correlation in multiple contexts. Based on the number of cancers with strong co-expression, miRNA pairs were classified as “ubiquitous,” “disease-specific,” or “other.” By intersecting the functional and expression-based classifications, we ultimately identified 12 ubiquitous and 33 disease-specific miRNA–miRNA pairs from the 169 predicted novel interactions. The mathematical form of classification based on function and expression was shown in the Supplementary Material Section 8. In light of the pronounced cancer-specific expression patterns among disease-specific pairs, we subsequently referred to them as “cancer-specific” pairs.

**Figure 5:**
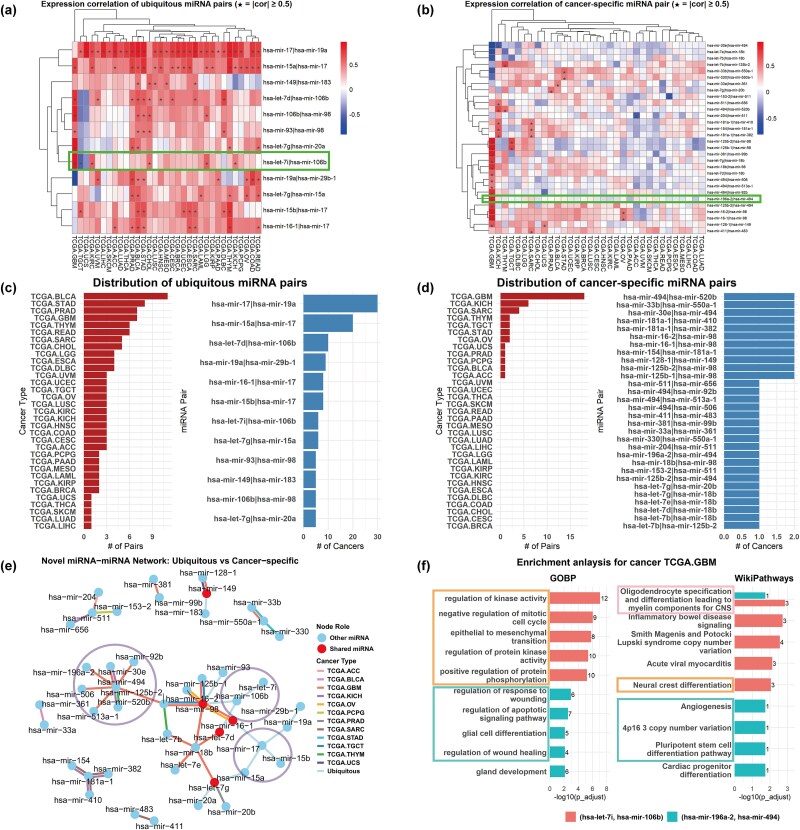
Exploration results of 30 cancer-specific miRNA pairs and 12 ubiquitous miRNA–miRNA pairs. (a)–(d) show the expression correlation patterns and distribution of these miRNA pairs across multiple cancer types. (e) presents the miRNA–miRNA functional interaction network composed of both ubiquitous and cancer-specific miRNA pairs. Shared miRNAs bridging both categories suggest partial functional overlap. (f) shows the top five terms and pathways from the enrichment analysis for the cancer-specific pair (hsa-mir-196a-2, hsa-mir-494) and the ubiquitous pair (hsa-let-7i, hsa-mir-106b), both of which were significantly co-expressed in GBM.

Figure 5(a)–(d) depicted the expression correlation and distribution of these classified miRNA pairs under 33 TCGA cancer types, in which cancer-specific miRNA pairs showed strong co-expression ($ |\textrm{cor}| \geq 0.5 $) in only one or two cancers, indicating high specificity and possible tumor-type-dependent regulation. These pairs were most frequently observed in glioblastoma multiforme (GBM). In contrast, ubiquitous pairs exhibited consistent and high correlation across diverse cancers, with several pairs recurring in over 20 cancer types. Figure 5(e) showed the miRNA–miRNA interaction network made up of both ubiquitous and cancer-specific miRNA pairs, exhibiting a modular and hierarchical structure. Ubiquitous pairs tended to form a dense and interconnected core, with hub miRNAs such as hsa-mir-106b and hsa-mir-17. In contrast, cancer-specific miRNA pairs were sparsely connected, forming smaller modules, indicative of more context-specific regulatory roles. Notably, hsa-mir-494 interacted with multiple other miRNAs (e.g. hsa-mir-196a-2) in GBM.

To investigate potential cooperative mechanisms, functional enrichment analysis was conducted on a cancer-specific pair (hsa-mir-196a-2, hsa-mir-494) and a ubiquitous pair (hsa-let-7i, hsa-mir-106b), both showing significant co-expression in GBM. Specifically, for each miRNA pair, we performed enrichment analysis (adjusted $P <0.05$) on target genes of both individual miRNAs, followed by identification of significantly co-enriched terms/pathways shared between the paired miRNAs. Figure 5(f) displays the top five overlapping enriched terms/pathways of these two pairs. It revealed that the ubiquitous miRNA pair (hsa-let-7i, hsa-mir-106b) was predominantly involved in core oncogenic processes such as kinase activity regulation, cell cycle control, and epithelial to mesenchymal transition, reflecting its pan-cancer relevance. In contrast, the cancer-specific pair (hsa-mir-196a-2, hsa-mir-494) was enriched in pathways related to glial cell differentiation, apoptotic signaling, and wound healing, highlighting its potential role in glioblastoma-specific biological regulation. These differences underscored the distinct functional contributions of ubiquitous versus cancer-specific miRNA–miRNA interactions.

### Case studies

#### Predicting novel miRNA–miRNA–disease associations

To demonstrate the capability of ConvNTC in predicting novel miRNA–miRNA–disease associations, we conducted case studies on Breast Neoplasms. Given a triple association $(m_{i},m_{j},d_{k})$, we validated it by verifying its pairwise associations i.e. $(m_{i},m_{j}),(m_{i},d_{k}),\\ (m_{j},d_{k})$. Specifically, we confirmed the miRNA–disease associations by querying the existing public databases, i.e. dbDEMC 2.0 [34], miRCancer [35], PhenomiR 2.0 [36], and HMDD v4.0 [37], and verified the miRNA–miRNA pairs using the TAM 2.0 [38]. Table 3 showed that all predicted top-10 miRNA pairs were confirmed.

**Table 3:** The detailed pieces of evidence of the top-10 predicted miRNA pairs for **breast neoplasms**

		MiRNA1-MiRNA2 pieces of evidence									
MiRNA1	MiRNA2	Enrichment Analysis using TAM 2.0 (-log10(p_value))							MiRNA1-Disease pieces of evidence	MiRNA2-Disease pieces of evidence	Triplets pieces of evidence
		Function	Disease	TF	Cluster	Family	TS	CS	Existing database	Existing database	Survival analysis (-log10(p_value))
**hsa-mir-125a**	**hsa-mir-99b**	2.39362	3.30437	3.57840	**5.70997**	4.73993	3.15366	2.95468	dbDEMC;miRCancer; PhenomiR;HMDDV4	dbDEMC;PhenomiR; HMDDV4	**3.25964**
hsa-mir-10a	hsa-mir-99b	0.00000	2.82136	3.57840	0.00000	**4.73993**	0.00000	0.00000	dbDEMC;miRCancer; PhenomiR;HMDDV4	dbDEMC;PhenomiR; HMDDV4	0.52288
hsa-mir-19b-1	hsa-mir-19b-2	2.64839	3.49975	3.73060	0.00000	**5.70997**	3.86646	3.11691	dbDEMC;PhenomiR	dbDEMC;PhenomiR	0.12494
**hsa-mir-99a**	**hsa-mir-99b**	3.82391	3.67684	0.00000	0.00000	**4.73993**	0.00000	0.00000	dbDEMC;miRCancer; PhenomiR;HMDDV4	dbDEMC;PhenomiR; HMDDV4	**1.50864**
hsa-mir-19a	hsa-mir-19b-2	2.64839	3.17420	3.73060	0.00000	**5.70997**	3.86646	3.11691	dbDEMC;miRCancer; PhenomiR;HMDDV4	dbDEMC;PhenomiR	0.43180
hsa-mir-181c	hsa-mir-181d	3.32808	2.93417	0.00000	**5.18709**	5.01100	0.00000	3.27327	dbDEMC;PhenomiR; HMDDV4	dbDEMC;PhenomiR; HMDDV4	1.22915
hsa-mir-154	hsa-mir-494	0.00000	2.55261	0.00000	3.25259	**3.95468**	0.00000	0.00000	dbDEMC;miRCancer; PhenomiR;HMDDV4	dbDEMC;miRCancer; PhenomiR;HMDDV4	0.21467
**hsa-mir-181a-2**	**hsa-mir-181d**	3.45204	2.92905	0.00000	0.00000	**5.01100**	0.00000	0.00000	dbDEMC;PhenomiR	dbDEMC;PhenomiR; HMDDV4	**2.16115**
hsa-mir-125b-2	hsa-mir-99b	2.39362	3.20235	0.00000	0.00000	**4.73993**	0.00000	0.00000	dbDEMC;PhenomiR; HMDDV4	dbDEMC;PhenomiR; HMDDV4	1.23657
hsa-mir-100	hsa-mir-99b	3.10876	3.89752	0.00000	0.00000	**4.73993**	0.00000	0.00000	dbDEMC;miRCancer; PhenomiR;HMDDV4	dbDEMC;PhenomiR; HMDDV4	0.50864

1 TAM 2.0 is a web tool for miRNA set enrichment analysis, which groups miRNAs into seven categories: miRNA-family sets, miRNA cluster sets, miRNA–disease, miRNA-function sets, miRNA-TF sets, tissue specificity sets, and cell specificity sets. Given a miRNA set, TAM 2.0 will enrich all miRNAs in this set across the above seven groups. First, the results of enrichment analysis for each miRNA pair (containing two miRNAs) in seven categories were obtained by TAM 2.0. Then, based on the condition that each term contained at least two miRNAs and $p\_value<0.05$, we believed that this term was co-enriched by two miRNAs in each miRNA pair. Finally, the average $-log10(p\_value)$ of all enriched terms by category was taken as the enrichment score of each miRNA pair in each category. 2 TF, TS, and CS are abbreviations for Transcription Factor, Tissue Specificity, and Cell Specificity, respectively.

To further evaluate the biological significance of triplet predictions, we performed survival analysis on top-10 predicted miRNA pairs using preprocessed miRNA expression and clinical data for breast cancer from The Cancer Genome Atlas (TCGA), obtained via the R package UCSCXenaTools [39]. The primary metric in our analysis was the risk score, constructed by multivariable Cox proportional hazards regression, based on both miRNA expression and clinical data. Kaplan–Meier survival analysis was then employed to assess the prognostic significance of these miRNA pairs. Table 3 showed that three miRNA pairs (hsa-mir-125a, hsa-mir-99b), (hsa-mir-99a, hsa-mir-99b), and (hsa-mir-181a-2, hsa-mir-181d) had a significant impact on the patient’s survival probability. Their survival curves and Pearson correlations of miRNA expression were shown in Supplementary Figure S2. For the pair (hsa-mir-125a, hsa-mir-99b), the combined analysis revealed more statistically significant distinction ($P<0.05$) between high-risk and low-risk groups compared with the individual analysis of hsa-mir-125a and hsa-mir-99b. Similar results were obtained for the other two miRNA pairs. These observations indicated that these miRNA pairs had a synergistic effect on breast cancer and exhibited promising prognostic value. Interestingly, the expressions of pairs (hsa-mir-125a, hsa-mir-99b) and (hsa-mir-181a-2, hsa-mir-181d) were positively correlated, whereas the pair (hsa-mir-99a, hsa-mir-99b) was negatively correlated, implying synergistic and antagonistic role of these miRNA pairs, respectively.

**Figure 6:**
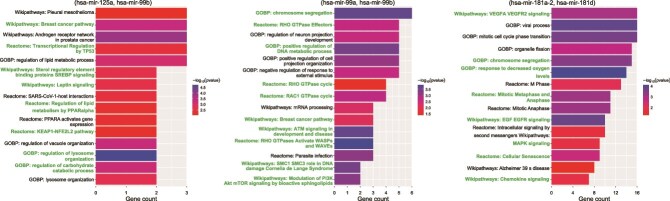
Enrichment analysis of the miRNA pairs (hsa-mir-125a, hsa-mir-99b), (hsa-mir-99a, hsa-mir-99b), and (hsa-mir-181a-2, hsa-mir-181d) related to breast neoplasms, based on GOBP, WikiPathways, and Reactome. Note that the green font indicates key processes and pathways associated with breast cancer.

Additionally, we performed enrichment analysis on these three miRNA pairs. For each pair, its gene set was composed of common target genes of each miRNA. The target genes of each pre-miRNA were defined as the union of target genes between its mature miRNAs from miRTarbase v9.0 [40]. We first used the R package clusterProfiler [41] and ReactomePA [42] to explore whether gene sets were involved in significant GOBP terms, Wikipathways, and Reactome pathways ($P <0.05$). Then, we compiled the top five enriched terms/pathways from GOBP, Wikipathways, and Reactome, and visualized them in Fig. 6 to highlight terms/pathways implicated in breast cancer progression. Enriched terms such as lysosome organization and chromosome segregation support cancer cell survival and genomic stability, while pathways like PI3K/Akt/mTOR and ATM signaling promote tumor growth and DNA repair. The breast cancer pathway specifically reflects hormonal and proliferative mechanisms, underscoring key processes in breast tumor progression.

#### Predicting novel synergistic drug pairs

We applied ConvNTC to predict novel synergistic drug pairs after training on the O’Neil dataset. We showcased the 855 potential drug pairs involving 42 drugs for malignant melanoma cancer cell line A375, reported in the paper [43]. The top-10 predicted drug combinations and their corresponding pieces of evidence were presented in Table 4. To assess the reliability of these predictions, we performed an extensive literature review and found that six out of the 10 predicted combinations were corroborated by prior studies or clinical trials. Confirmed Erlotinib-based combinations (e.g. with Crizotinib, Temsirolimus, Lenvatinib) enhanced anticancer efficacy by co-targeting EGFR, PI3K/mTOR, VEGFR, or c-MET pathways, thereby suppressing proliferation and inducing apoptosis. Other combinations (e.g. with axitinib, neratinib, erdafitinib) remained unvalidated in A375 cells but showed synergistic potential in other cancer types [44, 45]. These drug–drug–cell predictions exhibited promising therapeutic potential and warrant further validation, highlighting ConvNTC’s ability to identify clinically relevant synergistic drug combinations.

**Table 4:** The supporting evidence for the top 10 predicted drug combinations on the A375 cancer cell line

Drug A	Drug B	Cell line	Pieces of evidence
			Pubilications (PMID)
Erlotinib	Axitinib	A375	NA
Erlotinib	Temsirolimus	A375	24470557; 33159216
Erlotinib	Crizotinib	A375	27673365; 28961830; 31960422; 31078602
Erlotinib	Neratinib	A375	NA
Erlotinib	Lenvatinib	A375	36066408
Erlotinib	Erdafitinib	A375	NA
Erlotinib	Cabozantinib	A375	30915273
Erlotinib	Entrectinib	A375	NA
Erlotinib	Everolimus	A375	22378048; 22968184
Erlotinib	Copanlisib	A375	34734007

## Conclusion

Building on the premise of reformulating the A–A–B triplet prediction task as a third-order tensor completion problem, we presented a two-stage hybrid tensor completion framework ConvNTC to identify the interactions among molecules of the same type across various biological contexts. Different from prior work, ConvNTC modeled the tensor’s underlying multilinear structure by developing an MCTD method with three constraints. Furthermore, ConvNTC integrated CNN and FastKAN into a unified architecture, which could comprehensively capture the intrinsic high-order complexity of the tensor and the potential nonlinear interactions between objects.

Through comprehensive experiments on both MMD and DDC datasets, ConvNTC consistently outperformed existing linear and nonlinear methods across multiple evaluation settings, including A–A–B triplet prediction as well as derived A–A and A–B pairwise prediction tasks. The ablation studies further revealed the critical contributions of each architectural component, particularly the convolutional encoder and FastKAN predictor, which enabled ConvNTC to capture complex nonlinear dependencies while preserving the low-rank structure of the original tensor. Additionally, entity-level cross-validation experiments confirmed the model’s ability to generalize to novel diseases/cells and miRNAs/drugs, demonstrating its applicability in data-sparse biomedical scenarios. Moreover, analysis of the predicted miRNA–miRNA pairs, and case studies on the novel miRNA–miRNA–disease and drug–drug–cell triplet predictions illustrated the potential of ConvNTC in precision medicine and biomedical discovery, supporting its value as a powerful predictive tool in multi-relational biological data.

Despite the promising performance, our method has several limitations. First, it currently focuses only on a specific type of ternary relation (i.e. A–A–B), limiting its ability to model more complex heterogeneous triplets such as A–B–C. Second, the nonlinear module is sensitive to class imbalance, particularly in cases involving novel miRNA or drug entities. This is likely due to limited representation learning from initial features, which are derived solely from linear MCTD or randomly initialized embeddings. Such limitations may lead to information loss and noise. In future work, we plan to incorporate cross-attention mechanisms to better integrate linear features with raw similarity priors, thereby enhancing the robustness and expressiveness of learned representations.

**Key Points**

- We develop a convolutional neural tensor completion framework, termed ConvNTC, that collaboratively captures both multilinear and nonlinear relationships to predict “A–A–B” type triplets.
- ConvNTC is a two-stage deep computational model, which first employs multilinear tensor decomposition by integrating multiple constraints, and then develops a neural tensor completion method by combining CNN with KAN to capture the high-dimensional nonlinear interactions within tensor.
- Extensive experiments on three benchmark datasets demonstrate that ConvNTC outperforms various state-of-the-art baseline methods in triplet prediction. Case studies highlight ConvNTC’s ability to uncover biologically and clinically meaningful miRNA–miRNA–disease and drug–drug–cell triplets.
- ConvNTC is a versatile framework designed to explore interactions among molecules of the same type under different states/conditions, and this is the first study to introduce KANs into tensor completion.

## Supplementary Material

supplementary_file_bbaf372

## Data Availability

The source code is available at https://github.com/Liangyushi/ConvNTC.
