# Supplementary material for: ConvNTC: convolutional neural tensor completion for detecting “A–A–B” type biological triplets
Source: Brief Bioinform. 2025 Aug 1;26(4):bbaf372. doi: 10.1093/bib/bbaf372 (PMC12315554; doi:10.1093/bib/bbaf372)
Supplement: supplementary_file_bbaf372 [file supplementary_file_bbaf372.pdf]

Supplementary Materials

ConvNTC: Convolutional neural tensor completion for detecting 'A-A-B' type biological triplets

Pei Liu[1,2], Xiao Liang[1], Yue Li[2*], Jiawei Luo[1*]

[1]College of Computer Science and Electronic Engineering, Hunan University, Changsha, 410083, Hunan, China

[2]School of Computer Science, McGill University, Montreal, H3A 0C6, Quebec, Canada

[*]Corresponding author. yueli@cs.mcgill.ca and luojiawei@hnu.edu.cn

July 7, 2025

Contents

1 Supplementary Material Section 1: Pseudocode of MCTD 4

2 Supplementary Material Section 2: Structure of FastKAN 4

2.1 Kolmogorov-Arnold Network (KAN) 4

2.2 KAN with Radial Basis Function Networks 5

3 Supplementary Material Section 3: Dataset Description and Preprocessing 6

3.1 MMD dataset 6

3.1.1 Triplet relationship 6

3.1.2 Similarity matrices 7

3.2 DDC dataset 9

3.2.1 The O'Neil data 9

3.2.2 The NCI-ALMANAC data 10

4 Supplementary Material Section 4: Experimental setup 10

4.1 Dataset Division Strategy 10

4.2 Evaluation metrics 12

4.3 Baseline methods 13

4.3.1 Linear models 13

4.3.2 Nonlinear models 14

5 Supplementary Material Section 5: Parameters analysis 15

5.1 MCTD module 16

5.2 NTD module 16

6 Supplementary Material Section 6: Ablation study 17

7 Supplementary Material Section 7: Derivation of A–A and A–B Evaluation Sets from A–A–B Predictions 18

8 Supplementary Material Section 8: Classification of the novel miRNA-miRNA pairs 19

8.1 Function-based classification 19

8.2 Expression-based classification 20

List of Figures

S1 A Venn diagram comparing the edges in the known, predicted, and randomly generated miRNA–miRNA networks. 21

S2 The survival curves and expression correlations of the miRNA pairs (hsa-mir-125a, hsa-mir-99b), (hsa-mir-99a, hsa-mir-99b), and (hsa-mir-181a-2, hsa-mir-181d) in breast neoplasms. 24

List of Tables

S1 Summarization of ConvNTC, MCTD and other comparison methods. 15

S2 Hyperparameter settings for all methods. 16

S3 Summarization of ConvNTC and its variants. 18

S4 Performance comparison between ConvNTC and other baseline models on the DDC datasets (O'Neil and NCI-ALMANAC). Bold indicates the best performance; Underline indicates the second-best. 21

S5 Comparison results of all methods on the three datasets at the levels of new cell lines and new diseases, respectively. Bold indicates the best performance; Underline indicates the second-best. 22

S6 Comparison results of all methods on the three datasets at the levels of new miRNAs and new drugs. Bold indicates the best performance; Underline indicates the second-best. 23

1 Supplementary Material Section 1: Pseudocode of MCTD

The pseudocode of MCTD as follows:

Algorithm S1 The pseudocode of MCTD

Input: Triple relationship tensor $\mathcal{X}$, similarity matrices S_m, S_d, laplace matrices U, V calculated from S_m, S_d, and parameters $\mu, \eta, \alpha, \beta, \lambda$

Output: Reconstructed tensor $\mathcal{X}_{pre}$, learned factor matrices M, C, D

Initializing tensor $\mathcal{X}^0 = \mathcal{X}$, factor matrices M^0, C^0, D^0 by uniform distribution, other matrices and lagrange multipliers $H^0, Z^0, P^0, F^0, Y_1^0, Y_2^0, Y_3^0, R_1^0, R_2^0 = 0$, projection matrices $A_m^0 = 0, A_d^0 = 0$, and parameters $\rho_1^0, \rho_2^0, \rho_3^0, \theta_1^0, \theta_2^0 = 1e-6$, $t = 0, t_{max} = 100, tol = 1e-4$,

while $t < t_{max}$ **do**

  Updating A_m^{t+1}, A_d^{t+1} using $CG(S_m, M^t, M^t, \mu, \lambda)$ and $CG(S_d, D^t, D^t, \eta, \lambda)$ in **Eq.(16)**

  Updating $M^{t+1}, C^{t+1}, H^{t+1}, Z^{t+1}$ by **Eq.(3-6)**

  Updating $D^{t+1}, P^{t+1}, F^{t+1}$ by **Eq.(7-9)**

  Updating $Y_1^{t+1}, Y_2^{t+1}, Y_3^{t+1}, R_1^{t+1}, R_2^{t+1}$ and $\rho_1^{t+1}, \rho_2^{t+1}, \rho_3^{t+1}, \theta_1^{t+1}, \theta_2^{t+1}$ by **Eq.(10-14)**

  Updating $\mathcal{X}^{t+1}$ by **Eq.(17)**

  Calculating $err = \frac{\|\mathcal{X}^{t+1} - \mathcal{X}^t\|_F^2}{\mathcal{X}^t}$

  if $err < tol$ **then**

    break

  end if

end while

return $\mathcal{X}_{pre} = [\![M^{t+1}, C^{t+1}, D^{t+1}]\!]$, $M^{t+1}, C^{t+1}, D^{t+1}$

2 Supplementary Material Section 2: Structure of FastKAN

2.1 Kolmogorov-Arnold Network (KAN)

The key idea behind Kolmogorov-Arnold Network (KAN) proposed by Liu et al. (2024), is based on the Kolmogorov-Arnold representation theorem. This theorem allows a multivariate function $f(x)$ to be expressed as a superposition of simpler functions, which can be more easily learned by a neural network. Mathematically, for a smooth $f(x) : [0,1]^n \to \mathbb{R}$, it can be expressed as:

$$f(x) = \sum_{q}^{2n+1} \Phi_q \Big(\sum_{p}^{n} \phi_{q,p}(x[p]) \Big) \tag{1}$$

where Φ, ϕ are univariate non-linear functions with their own trainable parameters.

For a supervised learning task consisting of input-output pairs $(x[i], y[i])$, KAN aims to find f such that $y[i] \approx f(x[i])$ for all data points. In the original paper, KAN parameterizes each 1D function as a B-spline curve, with learnable coefficients of local B-spline basis functions. A general KAN network is a composition of L layers with shape $[n_0, n_1, ..., n_L]$: given an input vector $\boldsymbol{x_0} = [x[0], x[1], ..., x[n_0]] \in \mathbb{R}^{n_0}$ and the dimension

of output $n_L = 1$, the output is:

$$f(x) = \sum_{l_{L-1}=1}^{n_{L-1}} \phi_{L-1,l_L,l_L-1}\Big(\sum_{l_{L-2}=1}^{n_{L-2}} \cdots \Big(\sum_{l_2=1}^{n_2} \phi_{2,l_3,l_2}\Big(\sum_{l_1=1}^{n_1} \phi_{1,l_2,l_1}\Big(\sum_{l_0=1}^{n_0} \phi_{0,l_1,l_0}\Big(x_0[l_0]\Big)\Big)\Big)\Big)\cdots\Big) \tag{2}$$

where n_i is the number of nodes in the i-th layer, $x_0[l_0]$ is the l_0-th data point of $\boldsymbol{x_0}$ in the 0-th layer; $\phi_{l,i,j}$ is the activation function connecting the i-th neuron in the $(l+1)$-th layer and the j-th neuron in the l-th layer. More specifically, for each data point x in vector $\boldsymbol{x}$, the output of activation function $\phi(\cdot)$ in a single KAN layer is:

$$\begin{aligned} \phi(x) &= w_b b(x) + w_s spline(x) \\ b(x) &= SiLU(x) = \frac{x}{1+e^{(-x)}} \\ spline(x) &= \sum_i c_i B_i(x, n_g, k) \end{aligned} \tag{3}$$

where $b(\cdot)$ is the basis function similar to residual connections, $spline(\cdot)$ is defined as a linear combination of B-splines basis, and w_b, w_s are trainable factors to better control the overall magnitude of $b(\cdot)$, $spline(\cdot)$ functions; c_i is the learnable coefficient, $B_i(\cdot)$ is B-spline basis function, n_g is the number of spline grids, k is the order of B-spline basis. Original paper sets $n_g = 5, k = 3$, so the number of B-splines basis is $n_g + k = 8$ for each data point. The output of $\phi(\cdot)$ is the sum of the basis function $b(\cdot)$ and the spline function $spline(\cdot)$.

This hierarchical structure enables KANs to model complex functions by breaking them down into simpler components, which can be learned more efficiently by the neural network. However, it may still encounter efficiency bottlenecks due to B-spline functions.

2.2 KAN with Radial Basis Function Networks

FastKAN proposed by Li (2024), a new implementation of KANs that significantly accelerates the model calculation. Specifically, FastKAN employs Gaussian kernel-based radial basis functions (RBFs) to approximate 3rd-order B-spline basis. Furthermore, layer normalization is utilized to ensure the input remains within the RBF domain. These modifications result in a more streamlined implementation of FastKAN, while maintaining accuracy.

The fundamental concept behind radial basis functions (RBFs) is to approximate a target function by combining multiple radially symmetric functions, each centered at distinct points within the input space. The output of an RBF network is expressed as a linear combination of these radial basis functions, weighted by adjustable coefficients. Mathematically, for each data point x in vector $\boldsymbol{x}$, the Gaussian kernel-based

RBF network with n_g centers (i.e. each data point has n_g Gaussian kernels) can be represented as:

$$rbf(x) = \sum_{i=1}^{n_g} w_i \varphi\Big(\|x - c_i\|\Big)$$
$$\varphi(r) = exp\Big(-\frac{r^2}{2h^2}\Big) \tag{4}$$

where w_i are the adjustable weights or coefficients of the i-th center; $\varphi(\cdot)$ is the radial basis function with Gaussian kernel, which depends on the distance between the input x and a center c_i; r is the radial distance, and h is a parameter that controls the width or spread of the function. Therefore, for each data point x in vector $\boldsymbol{x}$, the output of the activation function $\phi(\cdot)$ in a FastKAN layer is:

$$\phi(x) = w_b b(x) + w_s\ rbf\Big(layernorm(x)\Big) \tag{5}$$

where $layernorm(\cdot)$ denotes layer normalization. In this paper, we set $n_g = 8$ as in the original paper.

3 Supplementary Material Section 3: Dataset Description and Preprocessing

3.1 MMD dataset

3.1.1 Triplet relationship

The miRNA–miRNA–disease (MMD) triplets are derived from our previous work (Liu et al., 2020), and are constructed based on 6,087 known miRNA–disease associations and 1,473 validated miRNA–miRNA interactions, encompassing 351 miRNAs from 193 families and 325 diseases. The principle for establishing each ternary relationship is as follows:

$$X(m_i, m_j, d_k) = \begin{cases} 1, < m_i, m_j >= 1, \ \ < m_i, d_k >= 1 \ and \ < m_j, d_k >= 1; \\ 0, otherwise. \end{cases} \tag{6}$$

where $X(m_i, m_j, d_k) = 1$ denotes that the miRNA pair (m_i, m_j) functions as a biomarker for disease d_k, implying that both m_i and m_j are associated with d_k and exhibit synergistic or functionally similar roles. Otherwise, $X(m_i, m_j, d_k) = 0$. The resulting third-order tensor $\mathcal{X} \in \mathbb{R}^{n_m \times n_m \times n_d}$ with the first two symmetric modes, where $n_m = 351$ and $n_d = 325$, is highly sparse, containing only 14,679 known interactions.

3.1.2 Similarity matrices

Mesh-based semantic similarity for disease. Based on the hierarchical directed acyclic graphs (DAGs), the disease semantic similarity is calculated by the way in Wang et al. (2010); Chen et al. (2018). The DAG of disease d consists of three elements, i.e., $DAG_d = (d, T_d, E_d)$, where T_d represents the node set of all ancestors of d including d, and E_d represents the edge set in DAG_d. Given disease d, its DAG_d and diseases k in DAG_d related to d, the contribution values $D_d^1(k)$ and $D_d^2(k)$ of disease k to semantic score of disease d are computed as follows:

$$
\begin{aligned}
D_{\mathrm{d}}^1(k) &= \begin{cases} 1, k = d. \\ \max\{\Delta \times D_{\mathrm{d}}^1(t) | t \in children \quad of \quad k\}, k \neq d. \end{cases} \\
D_d^2(k) &= -\log\left\{\frac{num(DAGs \quad including \quad k)}{num(all \quad diseases)}\right\}
\end{aligned}
\tag{7}
$$

where $\Delta = 0.5$ denotes the semantic contribution factor Xiao et al. (2018); Liang et al. (2019). The semantic value of disease d is computed as follows:

$$
DV_{type}(d) = \sum\nolimits_{k \in T_d} D_d^{type}(k) \quad s.t. \quad type = \{1, 2\} \tag{8}
$$

Based on the hypothesis that diseases with more shared nodes in their DAGs are more similar, two types of semantic similarity scores for each disease pair can be defined as below:

$$
DSS_{type}(d_i, d_j) = \frac{\sum_{k \in T_{d_i} \cap T_{d_j}} (D_{d_i}^{\mathrm{type}}(k) + D_{d_j}^{\mathrm{type}}(k))}{DV_{\mathrm{type}}(d_i) + DV_{\mathrm{type}}(d_j)} \tag{9}
$$

The disease semantic similarity can be calculated as below:

$$
DS_{semantic}(d_i, d_j) = (\sum\nolimits_{i \in type} DSS_i)/length(type) \tag{10}
$$

Sequence similarity for miRNA. As describe in Liang et al. (2019), the "pairwiseAlignment" function in the R package "Biostrings" is adopted to calculate the sequence similarity scores of miRNAs and build the similarity matrix MSS. Finally, the miRNA sequence simiarity matrix $A_{sequence}$ is obtained by the min-max normalization as follows:

$$
MS_{sequence}(m_i, m_j) = \frac{MSS(m_i, m_j) - MSS_{\min}}{MSS_{\max} - MSS_{\min}} \tag{11}
$$

where MSS_{max} and MSS_{min} represent the maximum and minimum values of the similarity matrix MSS.

Target-based functional similarity for miRNAs and diseases. Similar to the approach in Xiao et al. (2018), and based on the assumption that functionally similar miRNAs tend to regulate similar targets, and that diseases associated with similar targets are themselves likely to be similar, the gene similarity matrix $LLSN$ is derived by applying min–max normalization to the weighted gene association scores. Subsequently, the gene similarity scores related to miRNAs and diseases are computed as follows:

$$TTS_{data}(g_i, g_j) = \begin{cases} 0, & e(g_i, g_j) \notin LLSN \\ 1, & g_i = g_j \\ LLSN(g_i, g_j), & e(g_i, g_j) \in LLSN. \end{cases} \tag{12}$$
$$s.t. \quad g_i, g_j \in data, data = \{MT, DT\}$$

where $e(g_i, g_j)$ denotes the edge between gene g_i and g_j; MT and DT are miRNA-target associations and disease-gene associations, respectively. Consequently, the similarity score of each miRNA or disease pair is computed as follows:

$$TS_{data}(s_i, s_j) = \frac{\sum\limits_{g \in G_j} TTS_{data}(g, G_i) + \sum\limits_{g \in G_i} TTS_{data}(g, G_j)}{|G_i| + |G_j|} \tag{13}$$
$$MS_{target} = TS_{MT}, \quad DS_{target} = TS_{DT}$$

where G_i and G_j denote the gene sets related to item s_i and s_j (s is miRNA or disease), respectively; $|G_i|$ and $|G_j|$ denote the number of genes in G_i and G_j, respectively; $TTS_{data}(g, G_i) = \max\limits_{1 \le t \le k}(TTS_{data}(g, g_{it}))$ represents the contribution of gene g related to item s_j to the gene set G_i related to s_i.

Drug-based functional similarity for miRNAs and diseases. With the assumption that miRNAs with similar functions tend to be targeted by similar drugs, and diseases treated by similar drugs tend to be similar Chen et al. (2020); Luo et al. (2020), the drug similarity scores DrS based on the drug chemical structures were calculated by the way in Luo et al. (2017). Similar to target-based similarity, the drug similarity for miRNAs or diseases is computed as follows:

$$DRS_{data}(dr_i, dr_j) = \begin{cases} 0, & e(dr_i, dr_j) \notin DrS \\ 1, & dr_i = dr_j \\ DrS(dr_i, dr_j), & e(dr_i, dr_j) \in DrS. \end{cases} \tag{14}$$
$$s.t. \quad dr_i, dr_j \in data, data = \{MDrug, DDrug\}$$

where $e(dr_i, dr_j)$ denotes the edge between drug dr_i and dr_j; $MDrug$ and $DDrug$ are miRNA-drug associa-

tions and disease-drug associations, respectively. Finally, the drug-based similarity score of each miRNA or disease pair is computed as follows:

$$DrugS_{data}(s_i,s_j) = \frac{\sum\limits_{dr \in Dr_j} DRS_{data}(dr, Dr_i) + \sum\limits_{dr \in Dr_i} DRS_{data}(dr, Dr_j)}{|Dr_i| + |Dr_j|}$$
$$MS_{drug} = DrugS_{MDrug}, \quad DS_{drug} = DrugS_{DDrug} \tag{15}$$

where Dr_i and Dr_j denote the drug sets related to item s_i and s_j (s is miRNA or disease), respectively; $|Dr_i|$ and $|Dr_j|$ denote the number of drugs in Dr_i and Dr_j, respectively; $DRS_{data}(dr, Dr_i) = \max\limits_{1 \le t \le k}(DRS_{data}(dr, dr_{it}))$ represents the contribution of dr related to s_j to the drug set Dr_i related to s_i.

Finally, the miRNA and disease similarity matrices S_m, S_d are obtained using the following average operation:

$$S_m = avgerage(MS_{sequenence}, MS_{target}, MS_{drug})$$
$$S_d = avgerage(DS_{semantic}, DS_{target}, DS_{drug}) \tag{16}$$

3.2 DDC dataset

3.2.1 The O'Neil data

Triplet relationship. The O'Neil data (O'Neil et al., 2016) is downloaded from a previous study (Preuer et al., 2018), in which the synergistic effects of drug pairs are quantified using Loewe synergy scores. To ensure consistency between symmetric synergy records (d_1, d_2, c, s_1) and (d_2, d_1, c, s_2), we apply the operation $s = \max(s_1, s_2)$, such that the synergy scores for both edges (d_1, d_2, c) and (d_2, d_1, c) are unified, i.e., $s_1 = s_2 = s$. Subsequently, we obtain 45,474 DDC triplets with continuous synergy scores across 38 drugs and 39 cell lines.

Similarity matrices. The 4,387 chemical descriptors of drugs are gained by filtering out zero variance features, comprising 1,309 extended connectivity fingerprints with a radius of 6 (ECFP_6), 802 physicochemical, and 2,276 toxicophore-based binary features. The gene expression profiles of cell lines are sourced from the ArrayExpress database (accession number: E-MTAB-3610) (Iorio et al., 2016b). The raw microarray data is processed through quantile normalization and summarized using Factor Analysis for Robust Microarray Summarization (FARMS) (Hochreiter et al., 2006). Additionally, FARMS provides Informative/Non-Informative calls for each gene (Talloen et al., 2007), which are used to filter the expression matrix, resulting in the final 3,984 genomic features of cell lines. With the processed features, we use cosine method to obtain the similarity matrices for both drugs and cell lines.

3.2.2 The NCI-ALMANAC data

Triplet relationship. After downloading the preprocessed NCI-ALMANAC data from the referenced study (Zhao et al., 2024), in which the synergistic effects of drug pairs are quantified using ComboScores (Holbeck et al., 2017). Similarly to O'neil data, we apply the operation $s = \max(s_1, s_2)$ to ensure consistency between symmetric synergy records (d_1, d_2, c, s_1) and (d_2, d_1, c, s_2). Thus, we gain 148,278 DDC triplets involving 87 drugs and 55 cell lines.

Similarity matrices. The SMILES of the drugs are obtained from PubChem database (Kim et al., 2019). Based on the SMILES structures, we adopt the RDKit toolkit (Landrum et al., 2016) to compute the Tanimoto structure similarity for drugs. The gene expression profiles of cell lines are sourced from the Genomics of Drug Sensitivity in Cancer (GDSC) database (Yang et al., 2012), which characterizes and screens approximately 1,000 human cancer cell lines. Guided by the Library of Integrated Network-based Cellular Signatures (LINCS) project (Iorio et al., 2016a), a subset of 899 genes is selected to construct the gene expression feature for the cell lines. Based on the gene features of cell lines, we obtain the cell cosine similarity matrix by cosine method.

Since our objective is to explore synergistic interactions rather than the magnitude of synergy, we should binary the continuous synergy scores by a given threshold. Similar to (Preuer et al., 2018), we set the threshold to 30, to obtain highly synergistic drug combinations that are attractive for clinical studies. If the synergy score of a given drug pair exceeded 30, its synergy status is set to 1 (positive); otherwise, it is set to 0 (negative). After binarization, the O'Neil dataset contains 4,014 positive and 41,460 negative samples, while the NCI-ALMANAC dataset comprises 17,978 positive and 130,300 negative samples.

4 Supplementary Material Section 4: Experimental setup

To systematically assess the performance of ConvNTC, we conducted five iterations of five-fold cross-validation experiments on MMD and DDC datasets, and compared the results with several state-of-the-art methods.

4.1 Dataset Division Strategy

To ensure a robust and fair evaluation of the proposed model, we designed three distinct data partitioning strategies, all based on five-fold cross-validation. Each strategy aimed to test the model under different generalization settings while strictly preventing any form of information leakage.

- **Global MMD/DDC Triplet-Level Partition.** In the MMD datasets, we assumed that all missing

entries are negative samples. As a result, the number of negative examples significantly exceeds that of the positive ones. To address the imbalance between positive and negative samples, we randomly sampled a subset of negative examples equal in number to the positive samples from the unobserved entries (except in experiments specifically designed to analyze the effect of varying negative sample ratios).

After constructing a balanced dataset, we applied five-fold cross-validation. Both the positive and sampled negative samples were randomly divided into five equal subsets. In each fold, four subsets of positives and negatives were combined to form the training set, while the remaining subset served as the test set. This process was repeated five times to ensure that each subset was used exactly once as the test set. The final performance was reported as the average across all five folds.

For the DDC dataset, after binarizing the drug synergy scores, the ratio of positive to negative samples was approximately 1:10 for the O'Neil dataset and 1:8 for the NCI-ALMANAC dataset. Unlike MMD, we did not perform negative sample downsampling in this case. Instead, we directly conducted five-fold cross-validation on the full dataset. This design preserved the original class imbalance and allows for a more realistic evaluation of model performance under imbalanced conditions. The principles used to divide the DDC data were consistent with those applied to the MMD data.

- **Disease/Cell Line-Level Partition.** To evaluate the model's ability to generalize to unseen diseases or cell lines, we employed a five-fold cross-validation strategy with stratification based on disease/cell line identity. Specifically, all diseases (or cell lines) were randomly and exclusively assigned to one of the five folds. For each disease or cell line, we sampled negative miRNA–miRNA or drug–drug pairs equal in number to the positive associations involving that disease/cell line. This sampling was conducted independently for each disease/cell to maintain intra-disease balance.

  Consequently, all triplets—both positive and negative—were assigned to a fold based on the identity of their associated disease/cell line. This setting ensured that the model was evaluated on diseases or cells completely unseen during training. To ensure stability and reduce sampling bias, we repeated the splitting process under five different random seeds.

- **MiRNA/Drug-Level Partition.** To test generalization on unseen miRNAs or drugs, we designed a five-fold cross-validation scheme where each miRNA/drug was randomly assigned to a single fold. All triplets in which a given miRNA or drug appeared—either as the first or second entity—were grouped together. For example, if 'miRNA1' was assigned to the test set in Fold 1, then all associated edges—such as 'miRNA1–miRNA2–disease', 'miRNA2–miRNA1–disease', and 'miRNA3–miRNA1–disease'—were

also included in the test set to ensure a consistent and entity-level separation. For each miRNA/drug, a number of negative examples equal to the number of positives was randomly sampled.

During cross-validation, any sample (positive or negative) was assigned to a fold if at least one of its constituent miRNAs or drugs belonged to that fold. This guaranteed that test-fold entities were completely unseen during training. We repeated this process five times with different seeds for robustness.

To improve training efficiency for the framework comprising both linear and nonlinear components (e.g., ConvNTC, DTF, CTF-DDI), we employed a staged training strategy. Initially, we performed 5-fold cross-validation to train the linear model and obtained fold-specific linear representations. For each fold, the linear features used as input to the nonlinear model were generated from a linear model trained exclusively on the remaining four folds, thereby ensuring strict separation between training and testing data and avoiding any potential data leakage. Subsequently, to facilitate robust training of the nonlinear model, we further split each fold's training set by holding out 10% as a validation set. This validation subset was used exclusively for monitoring model convergence, enabling early stopping, and guiding model selection, without being used in gradient updates.

4.2 Evaluation metrics

We used eight standard supervised learning metrics to evaluate the performance of our proposed method. These include the Area Under Receiver Operating Characteristic curve (AUROC) and the Area Under Precision-Recall curve (AUPRC), which can quantify the predicting accuracy. In addition, we also utilize the following evaluation metrics to obtain a more comprehensive assessment:

$$\begin{aligned}
&Accuracy = \frac{TP + TN}{TP + TN + FP + FN}, \\
&Recall = \frac{TP}{TP + FN}, \quad Precision = \frac{TP}{TP + FP} \\
&F1 = \frac{2 \times Precision \times Recall}{Precision + Recall}, \quad Specificity = \frac{TN}{TN + FP}
\end{aligned} \tag{17}$$

where TP and TN represent the number of true positives and true negatives respectively, while FP and FN are false positives and false negatives, respectively. To statistically measure the significant improvement of performance between ConvNTC and baselines, we employed the paired t-test on these metrics at a significance level of 0.05.

4.3 Baseline methods

To ensure a comprehensive and fair evaluation, we selected a diverse set of baseline methods for comparison with ConvNTC. These baselines can be broadly categorized into linear models and nonlinear models, covering both classical tensor decomposition approaches and deep learning frameworks. All selected models were designed for handling third-order incomplete tensors, and were widely applied in related biomedical association prediction tasks.

4.3.1 Linear models

- CANDECOMP/PARAFAC (CP) (Kolda and Bader, 2009): A classical tensor factorization model without any auxiliary information which decomposes a tensor as a sum of rank-one tensors via alternating least squares (ALS) rules. The input structure of tensor is $A \times B \times C$, where A, B, C are three different objects.

- TFAI (Narita et al., 2012): A variant incorporates auxiliary information into the CP model by introducing graph Laplacian regularizations. The input structure of tensor is $A \times B \times C$.

- DrugCom (Chen and Li, 2018): A tensor completion method for capturing disease-related drug combinations using representation learning to integrate multiple sources of additional information about drugs and diseases. The input structure of tensor is $A \times A \times B$.

- miRCom (Liu et al., 2020): A novel tensor completion framework integrating multi-view miRNAs and diseases information for the discovery of potential disease-associated miRNA-miRNA pairs. The input structure of tensor is $A \times A \times B$.

- TDRC (Huang et al., 2021): A tensor decomposition method to predict multi-type miRNA-disease associations by constraining the factor matrices with auxiliary information of miRNAs and diseases. The input structure of tensor is $A \times B \times C$.

- CTF (Han et al., 2024): A constrained tensor decomposition model for predicting drug–drug–type triple relationship, incorporating drug similarity and other constraints into the CP model. The input structure of tensor is $A \times A \times B$.

The parameters except for r, of all baselines are set to the same values as those in original papers. Since the rank r is an intrinsic property of the tensor, we uniformly set $r = 57$ on MMD dataset, $r = 122$ on O'Neil data and $r = 57$ on NCI-ALMANAC data for all models that require rank r.

4.3.2 Nonlinear models

- DeepSynergy (Preuer et al., 2018): A deep learning approach for predicting the synergistic scores of drug combinations related to cell lines, consists of a normalization strategy to account for input data heterogeneity and a Multilayer Perceptron (MLP) with shape $[8182, 4096, 1]$ to model drug synergies. The input is the normalized feature of each known entry in a tensor with shape $A \times A \times B$.

- Costco (Liu et al., 2019): A versatile neural tensor completion model for sparse tensors, utilizing the expressive capabilities of convolutional neural networks (CNNs) to capture intricate nonlinear interactions within tensors. Its parameter-sharing strategy effectively maintains the desired low-rank structure. Furthermore, CoSTCo is scalable, as it avoids computationally or memory-intensive operations, such as the Kronecker product. The input is the index of each entry in tensor with with a shape of $A \times B \times C$, and the dimension of input features is .

- DTF (Sun et al., 2020): A deep tensor factorization model for predicting the synergy status of drug pairs related to cells, integrates a tensor factorization method CP_WOPT with a deep neural network (DNN). DTF mainly adopts CP_WOPT to generate the factors of each mode in tensor, and concentrates them to form the feature of each entry in tensor with shape $A \times A \times B$. Then, it normalizes the entry feature in a same way proposed in DeepSynergy, and feeds the normalized entry feature into MLP with shape $[2048, 1024, 512, 1]$ to calculate the predicted probabilities.

- GraphTF (Luo et al., 2021): A graph attention mechanism-based tensor decomposition method for predicting disease-associated miRNA-miRNA pairs. It adopts graph attention network to capture node features over multi-source biological network, and then uses the learned miRNA and disease features to reconstruct the association tensor via the Kronecker product. The inputs are two miRNA similarity matrices, one disease similarity matrix, and a tensor with a shape of $A \times B \times A$.

- CTF-DDI (Han et al., 2024): A novel methods for potential drug–drug interactions prediction combines CTF methods with a MLP layer ($[256, 256, 128, 1]$) to extract nonlinear features. It takes the learned tensor factors from CTF as the inputs, and outputs the enhanced predicted scores.

The hyperparameter settings for all aforementioned nonlinear methods were kept consistent with those reported in their original publications. Whenever available, we used the official implementations of these baseline models with their default configurations to ensure reproducibility. For methods without publicly available code, we re-implemented them faithfully based on the algorithmic descriptions provided in the original papers, using PyTorch as the backend framework. Specifically, DeepSynergy and DTF were re-

implemented in PyTorch, while DrugCom and miRCom were implemented in MATLAB, consistent with their original MATLAB-based implementations. Table S1 provides an overview of the proposed ConvNTC model and the selected baseline methods, while Table S2 presents a summary of their corresponding hyperparameter settings. For models requiring tensor rank parameters (e.g., CP, TFAI, TDRC), we set $r = 57$ on MMD dataset, $r = 122$ on O'Neil data and $r = 57$ on NCI-ALMANAC data across all applicable methods.

Table S1: Summarization of ConvNTC, MCTD and other comparison methods.

Methods	Tensor factorization	Auxilary information	Linear relation	Nonlinear relation
**ConvNTC (ours)**	✓	✓	✓	✓
**MCTD (ours)**	✓	✓	✓	−−
CP	✓	−−	✓	−−
TFAI	✓	✓	✓	−−
DrugCom	✓	✓	✓	−−
miRCom	✓	✓	✓	−−
TDRC	✓	✓	✓	−−
CTF	✓	✓	✓	−−
DeepSynergy	−−	✓	−−	✓
Costco	−−	−−	−−	✓
DTF	✓	−−	✓	✓
GraphTF	−−	✓	−−	✓
CTF-DDI	✓	✓	✓	✓

5 Supplementary Material Section 5: Parameters analysis

Considering that ConvNTC is a two-stage model comprising the MCTD and NTD modules, we first determined the optimal parameter combination for the six parameters (i.e., $r, \mu, \eta, \alpha, \beta, \lambda$) in MCTD module to streamline the search for the overall optimal configuration. Subsequently, with these six parameters fixed, we identified the optimal combination for the remaining five parameters (i.e., learning rate, $batch\_size, epoch, n_C, \gamma$) in the NTD module.

Table S2: Hyperparameter settings for all methods.

Method	r	μ	η	α	β	λ	lr	$batch\_size$	$epoch$	n_C	γ
**ConvNTC_1**	57	0.75	0.125	0.25	0.25	0.001	0.0001	256	500	114	0.5
**ConvNTC_2**	122	0.5	2	0.125	0.125	0.001	0.00001	256	500	244	0.8
**ConvNTC_3**	57	0.5	2	0.125	0.125	0.001	0.00001	512	500	114	0.8
**MCTD_1**	57	0.75	0.125	0.25	0.25	0.001	–	–	–	–	–
**MCTD_2**	122	0.5	2	0.125	0.125	0.001	–	–	–	–	–
**MCTD_3**	57	0.5	2	0.125	0.125	0.001	–	–	–	–	–
**CP**	57/122/57	–	–	–	–	–	–	–	–	–	–
**TFAI**	57/122/57	–	–	2	0.125	0.001	–	–	–	–	–
**DrugCom**	57/122/57	–	–	0.1	0.1	–	–	–	–	–	–
**miRCom**	57/122/57	–	–	1	0.1	–	–	–	–	–	–
**TDRC**	57/122/57	–	–	2	0.125	0.001	–	–	–	–	–
**CTF**	57/122/57	0.5	0.2	0.5	0.5	0.5	–	–	–	–	–
**DeepSynergy**	–	–	–	–	–	–	0.00001	64	1000	–	–
**Costco_1**	57	–	–	–	–	–	0.0001	256	500	114	–
**Costco_2**	122	–	–	–	–	–	0.00001	256	500	244	–
**Costco_3**	57	–	–	–	–	–	0.00001	512	500	114	–
**DTF**	57/1000/57	–	–	–	–	–	0.00001	128	1000	–	–
**GraphTF**	128/128/128	–	–	–	–	–	0.001	full-batch	300	–	–
**CTF-DDI**	57/122/57	0.5	0.2	0.5	0.5	0.5	0.0001	1000	300	–	–

[1] ConvNTC_1, ConvNTC_2, and ConvNTC_3 correspond to experiments conducted on the MMD, O'Neil, and NCI-ALMANAC datasets, respectively. Similarly, MCTD_1, MCTD_2, and MCTD_3, as well as Costco_1, Costco_2, and Costco_3, follow the same dataset assignments.
[2] For all baseline methods, the r values are listed in the format: MMD/O'Neil/NCI-ALMANAC dataset. For models requiring tensor rank parameters (e.g., CP, TFAI, TDRC), we set $r = 57$ on MMD dataset, $r = 122$ on O'Neil data and $r = 57$ on NCI-ALMANAC data across all applicable methods.
[3] For DTF, the rank for O'Neil is set to 1000 as the original paper.

5.1 MCTD module

Since λ had less influence on performance, we empirically set $\lambda = 0.001$. First, we fixed $r = 30$ on MMD dataset and $r = 32$ on DDC dataset (O'Neil and NCI-ALMANAC), and adopted grid search to evaluate performance of the MCTD module with parameters μ, η, α, β in ranges of $[2^{-3}, 2^{-2}, 2^{-1}, 0.75, 2^0, 2^1]$ on these three datasets. As a result, MCTD achieved the best performance on MMD when $\mu = 0.75, \eta = 0.125, \alpha = 0.25, \beta = 0.25$, and on both O'Neil and NCI-ALMANAC when $\mu = 0.5, \eta = 2, \alpha = 0.125, \beta = 0.125$. Using this optimal parameter combination, we assessed r in the range of $[30, ..., 127, 128]$ on MMD, and $[32, ..., 127, 128]$ on O'Neil and NCI-ALMANAC. To the end, MCTD achieved the best performance on MMD when $r = 57$, on O'Neil when $r = 122$, and on NCI-ALMANAC when $r = 57$.

5.2 NTD module

Since we designed an early stopping strategy during model training to prevent overfitting, the *epoch* was empirically set to 500. Based on the optimal parameter combination in MCTD module, we first fixed $\gamma = 0.5$, and adopted a grid search to assess the parameters lr (i.e., learning rate), $batch\_size, n_C$ in the ranges of

$[0.001, 0.0001, 0.00001]$, $[256, 512, 1024]$, $[\frac{1}{2}r, r, 2r]$ on MMD and DDC datasets. As a result, NTD achieved the best performance on MMD dataset when $lr = 0.0001$, $batch\_size = 256$, $n_C = 2r$, on O'Neil when $lr = 0.00001$, $batch\_size = 256$, $n_C = 2r$, and on NCI-ALMANAC when $lr = 0.00001$, $batch\_size = 512$, $n_C = 2r$. Based on the optimal combination of $lr, batch\_size, n_C$, we evaluated the influence of parameter γ in range of $[0, 0.1, 0.2, 0.3, 0.4, 0.5, 0.6, 0.7, 0.8, 0.9, 1]$ on these datasets. Finally, NTD achieved the best performance on MMD when $\gamma = 0.5$, on O'Neil and NCI-ALMANAC when $\gamma = 0.8$.

In conclusion, the optimal parameter setting of ConvNTC on MMD dataset was: $r = 57, \mu = 0.75, \eta = 0.125, \alpha = 0.25, \beta = 0.25, \lambda = 0.001, n_C = 2r, lr = 0.0001, batch\_size = 256, epoch = 500, \gamma = 0.5$. The best parameter setting of ConvNTC on O'Neil was: $r = 122, \mu = 0.5, \eta = 2, \alpha = 0.125, \beta = 0.125, \lambda = 0.001, n_C = 2r, lr = 0.00001, batch\_size = 256, epoch = 500, \gamma = 0.8$. The optimal parameter setting of ConvNTC on NCI-ALMANAC was: $r = 57, \mu = 0.5, \eta = 2, \alpha = 0.125, \beta = 0.125, \lambda = 0.001, n_C = 2r, lr = 0.00001, batch\_size = 512, epoch = 500, \gamma = 0.8$. Subsequent experiments were conducted under the optimal parameter combination for MMD and DDC dataset.

6 Supplementary Material Section 6: Ablation study

A summary of ConvNTC and its variants is provided in Supplementary Table S3. From the results of Table 1 in main paper, it is necessary to explain that on the DDC datasets, ConvNTC-mlp exhibited performance close to random guessing, with AUROC values around 0.5 and severely degraded F1 and Precision scores. We hypothesized that this was due to the severe class imbalance, where the ratio of positive to negative samples is close to 1:10 in the O'Neil data and close to 1:8 in the NCI-ALMANAC data. This imbalance likely caused the MLP-based predictor to overfit the majority class, leading to a skewed prediction distribution concentrated near zero. As a result, when applying the classification threshold, most predictions fell below the cutoff, yielding nearly all-negative predictions. This leads to inflated recall (1.0) when the threshold is zero and AUROC near 0.5, which reflects a complete loss of discriminative power.

Table S3: Summarization of ConvNTC and its variants.

Variants	MCTD	Index embedding	Factor emdedding	Convolutional encoder	Predictor
ConvNTC-nind	✓	$--$	✓	✓	$FastKAN$
ConvNTC-nfact	$--$	✓	✓	✓	$FastKAN$
ConvNTC-nconv	✓	✓	✓	$--$	$FastKAN$
ConvNTC-mlp	✓	✓	✓	✓	MLP
ConvNTC-nntd	✓	$-$	$--$	$--$	$--$
**ConvNTC**	✓	✓	✓	✓	$FastKAN$

[1] MCTD is a part of multi-linear relationship learning module; Embedding layer (including Index embedding and Factor embedding), Convolutional encoder and predictor is three parts of the nonlinear relationship learning module.

[2] ConvNTC-nind denotes we only use the predicting score derived from factor embedding to evaluate the impact of index embedding ($\gamma = 1$).

[3] ConvNTC-nfact denotes we only use the predicting score derived from index embedding to evaluate the impact of factor embedding ($\gamma = 0$).

[4] ConvNTC-nconv denotes the convolutional encoder is removed to assess the importance of it.

[5] ConvNTC-mlp denotes the FastKAN is replaced by MLP to evaluate the effectiveness of FastKAN.

[6] ConvNTC-nntd (i.e. MCTD module) denotes only the MCTD module is retained to obtain the predicting score to evaluate the impact of the whole nonlinear relationship learning moduled.

7 Supplementary Material Section 7: Derivation of A–A and A–B Evaluation Sets from A–A–B Predictions

To enable consistent and biologically meaningful evaluation across different relational levels, we decomposed the model's triplet-level predictions into two derived pairwise forms: **A–A** (miRNA–miRNA) and **A–B** (miRNA–disease). This approach ensured that all evaluations were conducted within a unified prediction space, facilitating fair comparison while avoiding the need to train separate models.

- **A–B Derivation (miRNA–disease)**: For each predicted triplet (m_1, m_2, d), two miRNA–disease pairs, (m_1, d) and (m_2, d), were extracted. Each pair inherited the same prediction score as the originating triplet. If a given miRNA–disease pair appeared in multiple triplets, the highest score was retained to reflect maximal predictive confidence.

- **A–A Derivation (miRNA–miRNA)**: The miRNA–miRNA pair (m_1, m_2) was extracted from each triplet independently of the disease context. When a miRNA pair was associated with multiple diseases

across different triplets, the maximum prediction score across all occurrences was similarly retained.

- **Label Construction for Evaluation**:

  Crucially, the ground-truth labels for A–A and A–B evaluations were not derived from the MMD triplet-level annotations. This was because a triplet labeled as negative (0) did not necessarily imply that both component associations (A–A and A–B) were absent. In many cases, a negative triplet arose due to the absence of only one underlying binary association.

  To avoid label noise and ensure reliable evaluation, the ground-truth labels for the A–A and A–B tasks were instead constructed from the original binary datasets: the MM dataset for miRNA–miRNA associations and the MD dataset for miRNA–disease associations. This strategy mitigated the risk of false negatives and prevented the loss of valid pairwise associations due to partial information at the triplet level.

- **Handling of Redundant Predictions**: To eliminate redundancy and emphasize confident predictions, scores for duplicate pairs were aggregated using the maximum value rather than averaging. This approach reflected the strongest model-supported evidence for each candidate association.

This unified decomposition and evaluation framework enabled a robust, fine-grained assessment of the model's ability to infer both higher-order and pairwise biological relationships, while maintaining consistency with the original triplet prediction outputs.

8 Supplementary Material Section 8: Classification of the novel miRNA-miRNA pairs

8.1 Function-based classification

To validate the biological significance of the predicted novel miRNA–miRNA pairs, we first performed a functional classification based on disease co-association and shared pathways across four annotation sources: Reactome, WikiPathway, GOBP, and disease annotations. Let d denote the number of shared diseases between a given pair of miRNAs, and let $\tilde{d}$ represent the median of all d values across the dataset. Additionally, let r, w, and g represent the number of shared pathways in Reactome, WikiPathway and GOBP, respectively. We first defined an indicator function $P = \mathbb{I}(r > 0 \vee w > 0 \vee g > 0)$ that captured whether the miRNA pair shares at least one functional pathway. Then, each miRNA pair was assigned to one of three categories as

follows:

$$L_{func} = \begin{cases} \text{"ubiquitous"}, & \text{if } d > \tilde{d} \text{ and } P = 1 \\ \text{"disease-specific"}, & \text{if } d \leq \tilde{d} \text{ and } P = 1 \\ \text{"other"}, & \text{if } P = 0 \end{cases}$$

This classification scheme distinguished miRNA interactions that were broadly involved in multiple diseases and pathways (ubiquitous), those associated with more restricted disease profiles (disease-specific), and pairs lacking known functional associations (other).

8.2 Expression-based classification

To enhance the biological significance of the above classification results, we defined the expression-based classification strategy. Based on the precursor miRNA expression profiles across 33 cancer types from TCGA via the UCSC database, we first computed the Pearson correlation coefficients between each pair. Then, we used the absolute correlation values to distinguish the miRNA pairs. Let $\mathbf{r} = (r_1, r_2, \ldots, r_n)$ denote the vector of Pearson correlation coefficients between a miRNA-miRNA pair across n different cancers, we defined the indicator function $high(r_i) = \mathbb{I}(|r_i| \geq \tau, \tau = 0.5)$. Let $H = \sum_{i=1}^{n} high(r_i)$ represent the total number of cancer types in which the miRNA pair exhibited high correlation, miRNA pairs were classified into three categories as follows:

$$L_{expr} = \begin{cases} \text{"ubiquitous"}, & \text{if } H \geq 5 \\ \text{"disease-specific"}, & \text{if } 1 \leq H \leq 2 \\ \text{"other"}, & \text{otherwise} \end{cases}$$

This classification reflected whether a miRNA pair showed consistent co-expression across many cancer types (ubiquitous), was co-expressed in only a few (disease-specific), or lacked strong correlation overall (other).

Table S4: Performance comparison between ConvNTC and other baseline models on the DDC datasets (O'Neil and NCI-ALMANAC). Bold indicates the best performance; Underline indicates the second-best.

Methods		AUPRC	AUROC	F1	Accuracy	Recall	Specificity	Precision	Paired t-test
**DDC dataset (O'Neil)**									
Linear	DrugCom	0.0627	0.3409	0.1234	0.3098	0.4655	0.2947	0.0766	8.6e-05
	CP	0.2289	0.6435	0.2754	0.8571	0.3067	0.9104	0.2544	0.00324
	TDRC	0.2917	0.6976	0.3364	0.8705	0.3718	0.9188	0.3106	0.00355
	miRCom	0.3639	0.8054	0.4171	0.8784	0.4920	0.9158	0.3655	0.00484
	TFAI	0.4383	0.8237	0.4643	0.8962	0.5093	0.9337	0.4295	0.00489
	CTF	0.6707	0.9273	0.6201	0.9330	0.6186	0.9635	0.6253	0.01002
	**MCTD (ours)**	**0.7625**	0.9498	**0.6997**	**0.9473**	**0.6953**	**0.9717**	**0.7054**	0.02280
Nonlinear	GraphTF	0.4061	0.8224	0.4307	0.8922	0.4622	0.9338	0.4064	0.00532
	DeepSynergy	0.4521	0.8517	0.4593	0.9011	0.4753	0.9423	0.4474	0.00628
	Costco	0.6027	0.8626	0.5686	0.8914	0.6167	0.918	0.5545	0.00035
	CTFDDI	0.6444	0.9174	0.5967	0.9297	0.5893	0.9626	0.6073	0.00994
	DTF	0.6850	0.9408	0.6352	0.9338	0.6529	0.9609	0.6207	0.00907
	**ConvNTC (ours)**	0.7286	**0.9503**	0.6673	0.9402	0.6795	0.9654	0.6583	
**DDC dataset (NCI-ALMANAC)**									
Linear	DrugCom	0.2070	0.7108	0.3283	0.6686	**0.6623**	0.6694	0.2193	0.01996
	miRCom	0.2314	0.7346	0.3464	0.7198	0.6119	0.7347	0.2420	0.01537
	TDRC	0.5341	0.8288	0.5156	0.8814	0.5206	0.9312	0.5128	0.02642
	CTF	0.5578	0.8389	0.5302	0.8870	0.5259	0.9368	0.5359	0.21870
	CP	0.5598	0.8378	0.5341	0.8879	0.5303	**0.9372**	0.5390	0.12953
	TFAI	0.5646	0.8390	0.5356	0.8876	0.5348	0.9362	0.5372	0.06651
	**MCTD (ours)**	**0.5772**	**0.8503**	**0.5437**	**0.8891**	0.5446	0.9367	**0.5445**	0.00794
Nonlinear	DeepSynergy	0.3062	0.7401	0.3708	0.8030	0.4787	0.8478	0.3034	0.00312
	GraphTF	0.3436	0.7433	0.3812	0.8182	0.4613	0.8674	0.3270	0.00209
	Costco	0.4544	0.7440	0.4214	0.7719	0.5329	0.8049	0.3951	0.00151
	CTFDDI	0.5247	0.8300	0.5025	0.8767	0.5135	0.9268	0.4931	0.00248
	DTF	0.5264	0.8358	0.5033	0.8743	0.5250	0.9225	0.4845	0.00880
	**ConvNTC (ours)**	0.5530	0.8475	0.5220	0.8829	0.5274	0.9320	0.5179	

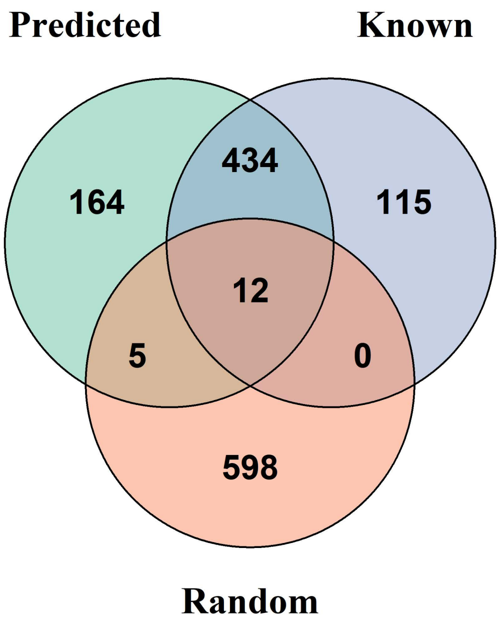

Figure S1: A Venn diagram comparing the edges in the known, predicted, and randomly generated miRNA–miRNA networks.

Table S5: Comparison results of all methods on the three datasets at the levels of new cell lines and new diseases, respectively. Bold indicates the best performance; Underline indicates the second-best.

Methods		AUPRC	AUROC	F1	Accuracy	Recall	Specificity	Precision	Paired t-test
**MMD dataset: New disease**									
Linear	TDRC	0.7500	0.5000	0.6667	0.5000	**1.0000**	0.0000	0.5000	0.01034
	TFAI	0.7500	0.5000	0.6667	0.5000	**1.0000**	0.0000	0.5000	0.01034
	CP	0.7500	0.5000	0.6667	0.5000	**1.0000**	0.0000	0.5000	0.01034
	CTF	0.7500	0.5000	0.6667	0.5000	**1.0000**	0.0000	0.5000	0.01034
	miRCom	0.8284	0.7427	0.7482	0.7644	0.6965	0.8323	0.8234	3.1e-05
	**MCTD (ours)**	0.9177	0.8788	0.8375	0.853	0.7594	0.9467	0.9352	0.00313
	DrugCom	0.9509	0.9477	0.9179	0.9053	0.9356	0.8751	0.9141	0.00040
Nonlinear	Costco	0.9116	0.8923	0.8722	0.8515	0.9185	0.7844	0.8463	0.00046
	GraphTF	0.8934	0.9212	0.8670	0.8608	0.9069	0.8147	0.8307	0.00019
	CTFDDI	0.9435	0.9296	0.8651	0.8731	0.8197	0.9265	0.9183	0.00101
	DeepSynergy	0.9609	0.9580	0.8988	0.8989	0.8984	0.8993	0.8994	0.00030
	DTF	0.9705	0.9642	0.9231	0.9244	0.9083	0.9405	0.9385	0.00032
	**ConvNTC (ours)**	**0.9941**	**0.9942**	**0.9851**	**0.9852**	0.9819	**0.9884**	**0.9884**	
**DDC dataset (O'Neil): New cell**									
Linear	DrugCom	0.0568	0.3426	0.1232	0.2258	0.5706	0.1932	0.0696	0.00542
	TDRC	0.5442	0.5000	0.1621	0.0884	**1.0000**	0.0000	0.0884	0.15184
	TFAI	0.5442	0.5000	0.1621	0.0884	**1.0000**	0.0000	0.0884	0.15184
	CP	0.5442	0.5000	0.1621	0.0884	**1.0000**	0.0000	0.0884	0.15184
	CTF	0.5442	0.5000	0.1621	0.0884	**1.0000**	0.0000	0.0884	0.15184
	miRCom	0.1744	0.6142	0.2414	0.8119	0.3310	0.8575	0.2002	0.00088
	**MCTD (ours)**	0.3154	0.7113	0.3700	0.8777	0.4067	0.9230	0.3459	0.00932
Nonlinear	CTFDDI	0.3036	0.7089	0.3256	0.8771	0.3299	0.9292	0.3406	0.01213
	Costco	0.3383	0.7233	0.3719	0.7834	0.5188	0.8097	0.3324	0.00165
	DeepSynergy	0.2985	0.7610	0.3425	0.8517	0.4331	0.8917	0.2913	0.00141
	GraphTF	0.4079	0.8224	0.4301	**0.8957**	0.4476	**0.9385**	0.4182	0.15181
	DTF	**0.4576**	**0.8544**	**0.4600**	0.8933	0.5121	0.9300	**0.4237**	0.05275
	**ConvNTC (ours)**	0.4477	0.8478	0.4550	0.8898	0.5173	0.9255	0.4090	
**DDC dataset (NCI-ALMANAC): New cell**									
Linear	TDRC	**0.5607**	0.5000	0.2163	0.1214	**1.0000**	0.0000	0.1214	0.25640
	TFAI	**0.5607**	0.5000	0.2163	0.1214	**1.0000**	0.0000	0.1214	0.25640
	CP	**0.5607**	0.5000	0.2163	0.1214	**1.0000**	0.0000	0.1214	0.25640
	CTF	**0.5607**	0.5000	0.2163	0.1214	**1.0000**	0.0000	0.1214	0.25640
	miRCom	0.1266	0.5188	0.2202	0.2447	0.8782	0.1567	0.1263	0.10960
	DrugCom	0.1573	0.6106	0.2565	0.5345	0.6600	0.5170	0.1598	0.05730
	**MCTD (ours)**	0.3236	0.6759	0.3547	0.8354	0.3718	0.8992	0.3426	0.00200
Nonlinear	CTFDDI	0.3281	0.6615	0.3414	0.8487	0.3223	**0.9211**	0.3693	0.00840
	Costco	0.3531	0.6826	0.3494	0.7505	0.4638	0.7905	0.3236	0.01870
	DeepSynergy	0.2880	0.7149	0.3526	0.7940	0.4618	0.8394	0.2881	0.02750
	GraphTF	0.3435	0.7429	0.3807	0.8242	0.4458	0.8762	0.3340	0.04680
	**DTF**	0.4206	**0.7619**	**0.4178**	0.8569	0.4233	0.9165	0.4158	0.49000
	**ConvNTC (ours)**	0.4173	0.7583	0.4157	**0.8586**	0.4138	0.9199	**0.4199**	

Table S6: Comparison results of all methods on the three datasets at the levels of new miRNAs and new drugs. Bold indicates the best performance; Underline indicates the second-best.

Methods		AUPRC	AUROC	F1	Accuracy	Recall	Specificity	Precision	Paired t-test
**MMD dataset: New miRNA**									
	miRCom	0.6094	0.4911	0.6165	0.4461	0.9979	0.0001	0.4465	0.05108
	CP	0.7231	0.5000	0.6167	0.4463	**1.0000**	0.0000	0.4463	0.07324
	DrugCom	0.4728	0.5578	0.6198	0.4560	0.9938	0.0212	0.4509	0.04346
Linear	TDRC	0.7523	0.6933	0.6745	0.7243	0.6367	0.7917	0.7305	0.93519
	**MCTD (ours)**	0.8259	0.8019	0.7332	0.7653	0.7244	0.7977	0.7444	0.24945
	CTF	0.8978	0.8952	0.8277	0.8434	0.8474	0.8402	0.8096	0.00521
	TFAI	0.8962	0.9126	0.8372	0.8464	0.8893	0.8118	0.7913	0.00100
	Costco	0.5879	0.6805	0.6464	0.6465	0.7148	0.6003	0.6177	0.04628
	DTF	0.8065	0.8421	0.7520	0.7647	0.8020	0.7333	0.7084	0.06483
Nonlinear	CTFDDI	0.9104	0.9280	0.8577	0.8600	0.9043	0.8224	0.8216	0.00055
	GraphTF	0.9096	0.9412	0.8686	0.8776	0.9066	0.8537	0.8339	0.00078
	DeepSynergy	**0.9498**	**0.9588**	**0.8834**	**0.8946**	0.8983	**0.8915**	**0.8692**	0.00092
	**ConvNTC (ours)**	0.7982	0.8044	0.7101	0.6931	0.8377	0.5722	0.6190	
**DDC dataset (O' Neil): New drug**									
	DrugCom	0.0794	0.4124	0.1353	0.3786	0.4985	0.3580	0.0820	0.01696
	CP	0.5439	0.5000	0.1602	0.0879	**1.0000**	0.0000	0.0879	0.34943
	TDRC	0.1589	0.5594	0.2056	0.7083	0.3932	0.7214	0.1624	0.00174
Linear	miRCom	0.1665	0.5937	0.2135	0.7536	0.3532	0.7791	0.1767	0.00021
	**MCTD (ours)**	0.2440	0.6326	0.2815	0.8545	0.3047	0.9034	0.2763	0.04992
	TFAI	0.2592	0.6636	0.2952	0.8511	0.3396	0.8974	0.2729	0.03570
	CTF	0.2914	0.6937	0.3267	0.8657	0.3595	0.9118	0.3086	0.63455
	Costco	0.2143	0.6350	0.2592	0.8104	0.3481	0.8538	0.2288	0.00071
	DeepSynergy	0.2283	0.7039	0.2902	0.8317	0.3818	0.8716	0.2411	0.00334
Nonlinear	CTFDDI	0.3009	0.7381	0.3306	0.8328	0.4119	0.8704	0.3105	0.97335
	DTF	0.3117	0.7629	0.3387	0.8548	0.4121	0.8933	0.3008	0.01082
	GraphTF	**0.3934**	**0.8234**	**0.4197**	**0.8919**	0.4564	**0.9315**	**0.3928**	0.00038
	**ConvNTC (ours)**	0.2941	0.7478	0.3249	0.8531	0.3894	0.8938	0.2904	
**DDC dataset (NCI-ALMANAC): New drug**									
	CP	**0.5609**	0.5000	0.2170	0.1218	**1.0000**	0.0000	0.1218	0.59285
	DrugCom	0.1228	0.5022	0.2177	0.1540	0.9689	0.0410	0.1228	0.33298
	miRCom	0.1368	0.5282	0.2207	0.2173	0.9115	0.1213	0.1258	0.33670
Linear	TDRC	0.1519	0.5414	0.2235	0.3800	0.7243	0.3263	0.1376	0.25045
	**MCTD (ours)**	0.1628	0.5605	0.2314	0.5230	0.5806	0.5122	0.1503	0.13687
	CTF	0.1854	0.5870	0.2541	0.6934	0.4245	0.7299	0.1851	0.04735
	TFAI	0.1955	0.5876	0.2578	0.6930	0.4274	0.7285	0.1920	0.08023
	Costco	0.1413	0.5815	0.2430	0.5755	0.5512	0.5788	0.1594	0.10872
	DTF	0.2019	0.6359	0.2720	0.6912	0.4728	0.7211	0.1920	0.34252
Nonlinear	CTFDDI	0.2602	0.6562	0.3106	0.7763	0.4103	0.8262	0.2561	0.07202
	DeepSynergy	0.2482	0.6829	0.3175	0.7598	0.4549	0.8015	0.2471	0.00613
	GraphTF	0.3437	**0.7429**	**0.3802**	**0.8173**	0.4611	**0.8664**	**0.3245**	0.00164
	**ConvNTC (ours)**	0.2069	0.6350	0.2729	0.6936	0.4685	0.7237	0.1945	

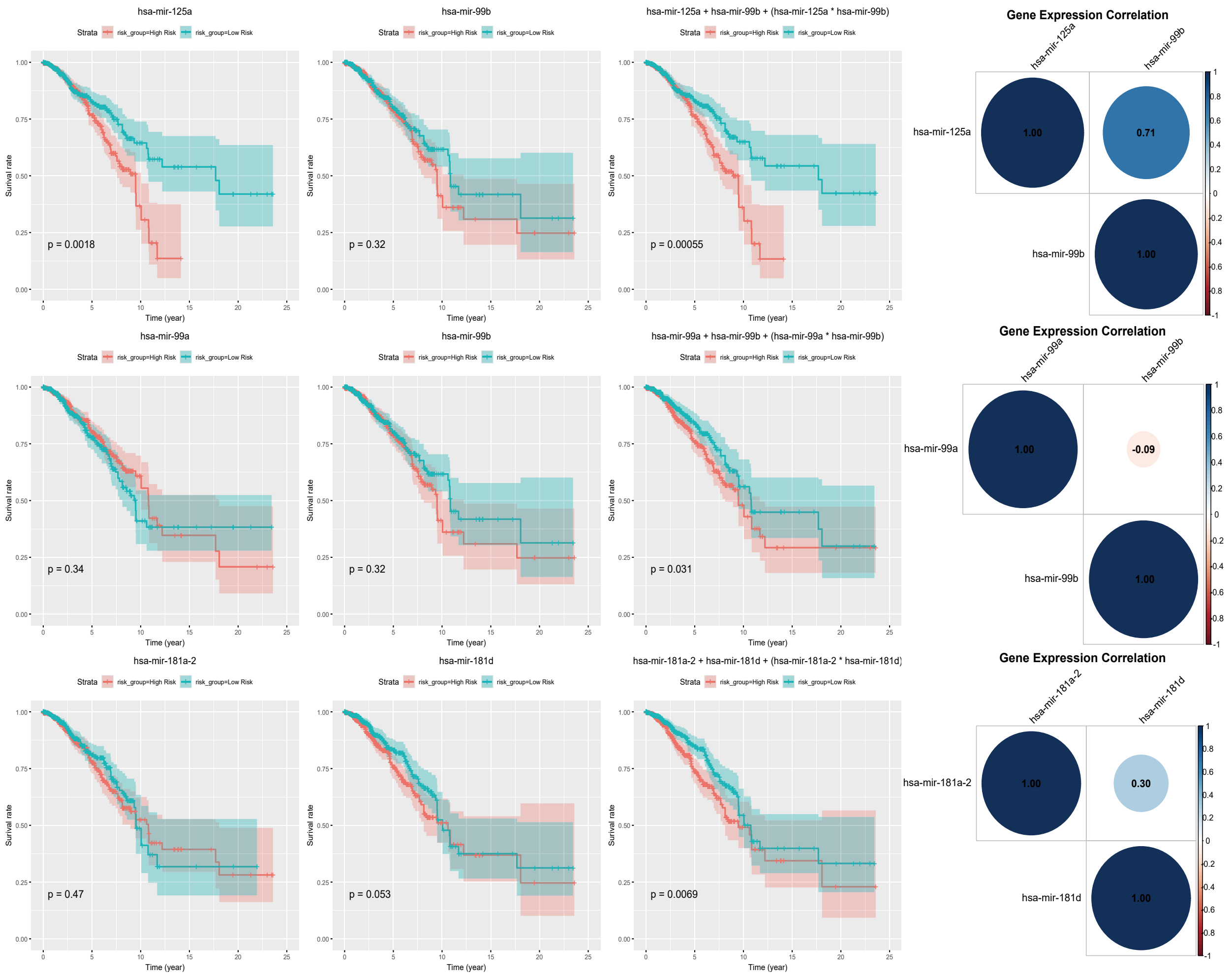

Figure S2: The survival curves and expression correlations of the miRNA pairs (hsa-mir-125a, hsa-mir-99b), (hsa-mir-99a, hsa-mir-99b), and (hsa-mir-181a-2, hsa-mir-181d) in breast neoplasms.

References

H. Chen and J. Li. Drugcom: Synergistic discovery of drug combinations using tensor decomposition. In *2018 IEEE International Conference on Data Mining (ICDM)*, pages 899–904. IEEE, 2018.

X. Chen et al. Predicting mirna–disease association based on inductive matrix completion. *Bioinformatics*, 34(24):4256–4265, 2018. ISSN 1367-4803.

X. Chen et al. Microrna-small molecule association identification: from experimental results to computational models. *Briefings in bioinformatics*, 21(1):47–61, 2020. ISSN 1467-5463.

G. Han, L. Peng, A. Ding, Y. Zhang, and X. Lin. Ctf-ddi: Constrained tensor factorization for drug–drug interactions prediction. *Future Generation Computer Systems*, 161:26–34, 2024.

S. Hochreiter, D.-A. Clevert, and K. Obermayer. A new summarization method for affymetrix probe level data. *Bioinformatics*, 22(8):943–949, 2006.

S. L. Holbeck, R. Camalier, J. A. Crowell, J. P. Govindharajulu, M. Hollingshead, L. W. Anderson, E. Polley, L. Rubinstein, A. Srivastava, D. Wilsker, et al. The national cancer institute almanac: a comprehensive screening resource for the detection of anticancer drug pairs with enhanced therapeutic activity. *Cancer research*, 77(13):3564–3576, 2017.

F. Huang, X. Yue, Z. Xiong, Z. Yu, S. Liu, and W. Zhang. Tensor decomposition with relational constraints for predicting multiple types of microrna-disease associations. *Briefings in bioinformatics*, 22(3):bbaa140, 2021.

F. Iorio, T. A. Knijnenburg, D. J. Vis, G. R. Bignell, M. P. Menden, M. Schubert, N. Aben, E. Gonçalves, S. Barthorpe, H. Lightfoot, et al. A landscape of pharmacogenomic interactions in cancer. *Cell*, 166(3): 740–754, 2016a.

F. Iorio, T. A. Knijnenburg, D. J. Vis, G. R. Bignell, M. P. Menden, M. Schubert, N. Aben, E. Gonçalves, S. Barthorpe, H. Lightfoot, et al. A landscape of pharmacogenomic interactions in cancer. *Cell*, 166(3): 740–754, 2016b.

S. Kim, J. Chen, T. Cheng, A. Gindulyte, J. He, S. He, Q. Li, B. A. Shoemaker, P. A. Thiessen, B. Yu, et al. Pubchem 2019 update: improved access to chemical data. *Nucleic acids research*, 47(D1):D1102–D1109, 2019.

T. G. Kolda and B. W. Bader. Tensor decompositions and applications. *SIAM review*, 51(3):455–500, 2009.

G. Landrum et al. Rdkit: Open-source cheminformatics software, 2016.

Z. Li. Kolmogorov-arnold networks are radial basis function networks. *arXiv preprint arXiv:2405.06721*, 2024.

C. Liang, S. Yu, and J. Luo. Adaptive multi-view multi-label learning for identifying disease-associated candidate mirnas. *PLoS computational biology*, 15(4):e1006931, 2019. ISSN 1553-7358.

H. Liu, Y. Li, M. Tsang, and Y. Liu. Costco: A neural tensor completion model for sparse tensors. In *Proceedings of the 25th ACM SIGKDD International Conference on Knowledge Discovery & Data Mining*, pages 324–334, 2019.

P. Liu, J. Luo, and X. Chen. mircom: tensor completion integrating multi-view information to deduce the potential disease-related mirna-mirna pairs. *IEEE/ACM Transactions on Computational Biology and Bioinformatics*, 19(3):1747–1759, 2020.

Z. Liu, Y. Wang, S. Vaidya, F. Ruehle, J. Halverson, M. Soljačić, T. Y. Hou, and M. Tegmark. Kan: Kolmogorov-arnold networks. *arXiv preprint arXiv:2404.19756*, 2024.

J. Luo, Z. Lai, C. Shen, P. Liu, and H. Shi. Graph attention mechanism-based deep tensor factorization for predicting disease-associated mirna-mirna pairs. In *2021 IEEE International Conference on Bioinformatics and Biomedicine (BIBM)*, pages 189–196. IEEE, 2021.

J. Luo et al. Incorporating clinical, chemical and biological information for predicting small molecule-microrna associations based on non-negative matrix factorization. *IEEE/ACM Transactions on Computational Biology and Bioinformatics*, 2020. ISSN 1545-5963.

Y. Luo et al. A network integration approach for drug-target interaction prediction and computational drug repositioning from heterogeneous information. *Nature communications*, 8(1):1–13, 2017. ISSN 2041-1723.

A. Narita, K. Hayashi, R. Tomioka, and H. Kashima. Tensor factorization using auxiliary information. *Data Mining and Knowledge Discovery*, 25:298–324, 2012.

J. O'Neil, Y. Benita, I. Feldman, M. Chenard, B. Roberts, Y. Liu, J. Li, A. Kral, S. Lejnine, A. Loboda, et al. An unbiased oncology compound screen to identify novel combination strategies. *Molecular cancer therapeutics*, 15(6):1155–1162, 2016.

K. Preuer, R. P. Lewis, S. Hochreiter, A. Bender, K. C. Bulusu, and G. Klambauer. Deepsynergy: predicting anti-cancer drug synergy with deep learning. *Bioinformatics*, 34(9):1538–1546, 2018.

Z. Sun, S. Huang, P. Jiang, and P. Hu. Dtf: deep tensor factorization for predicting anticancer drug synergy. *Bioinformatics*, 36(16):4483–4489, 2020.

W. Talloen, D.-A. Clevert, S. Hochreiter, D. Amaratunga, L. Bijnens, S. Kass, and H. W. Göhlmann. I/ni-calls for the exclusion of non-informative genes: a highly effective filtering tool for microarray data. *Bioinformatics*, 23(21):2897–2902, 2007.

D. Wang et al. Inferring the human microrna functional similarity and functional network based on microrna-associated diseases. *Bioinformatics*, 26(13):1644–1650, 2010. ISSN 1460-2059.

Q. Xiao et al. A graph regularized non-negative matrix factorization method for identifying microrna-disease associations. *Bioinformatics*, 34(2):239–248, 2018.

W. Yang, J. Soares, P. Greninger, E. J. Edelman, H. Lightfoot, S. Forbes, N. Bindal, D. Beare, J. A. Smith, I. R. Thompson, et al. Genomics of drug sensitivity in cancer (gdsc): a resource for therapeutic biomarker discovery in cancer cells. *Nucleic acids research*, 41(D1):D955–D961, 2012.

X. Zhao, J. Xu, Y. Shui, M. Xu, J. Hu, X. Liu, K. Che, J. Wang, and Y. Liu. Permutedds: a permutable feature fusion network for drug-drug synergy prediction. *Journal of Cheminformatics*, 16(1):41, 2024.
